# Exploring the potential mechanisms of Tongmai Jiangtang capsules in treating diabetic nephropathy through multi-dimensional data

**DOI:** 10.3389/fendo.2023.1172226

**Published:** 2023-11-01

**Authors:** Yi Liu, Xin Cui, Xuming Zhang, Zhuoting Xie, Weili Wang, Junyu Xi, Yanming Xie

**Affiliations:** ^1^ Institute Of Basic Research In Clinical Medicine, China Academy Of Chinese Medical Sciences, Beijing, China; ^2^ Department of Orthopedics, The Second Hospital of Jilin University, Changchun, Jilin, China

**Keywords:** Tongmai Jiangtang capsule (TMJT), Traditional Chinese Medicine, bioinformatics, machine learning, nomogram, network pharmacology, molecular docking, Mendelian randomization (MR)

## Abstract

**Background:**

Diabetic nephropathy (DN) is a prevalent and debilitating disease that represents the leading cause of chronic kidney disease which imposes public health challenges Tongmai Jiangtang capsule (TMJT) is commonly used for the treatment of DN, albeit its underlying mechanisms of action are still elusive.

**Methods:**

This study retrieved databases to identify the components and collect the targets of TMJT and DN. Target networks were constructed to screen the core components and targets. Samples from the GEO database were utilized to perform analyses of targets and immune cells and obtain significantly differentially expressed core genes (SDECGs). We also selected a machine learning model to screen the feature genes and construct a nomogram. Furthermore, molecular docking, another GEO dataset, and Mendelian randomization (MR) were utilized for preliminary validation. We subsequently clustered the samples based on SDECG expression and consensus clustering and performed analyses between the clusters. Finally, we scored the SDECG score and analyzed the differences between clusters.

**Results:**

This study identified 13 SDECGs between DN and normal groups which positively regulated immune cells. We also identified five feature genes (*CD40LG*, *EP300*, *IL1B*, *GAPDH*, and *EGF*) which were used to construct a nomogram. MR analysis indicated a causal link between elevated IL1B levels and an increased risk of DN. Clustering analysis divided DN samples into four groups, among which, C1 and CI were mainly highly expressed and most immune cells were up-regulated. C2 and CII were the opposite. Finally, we found significant differences in SDECG scores between C1 and C2, CI and CII, respectively.

**Conclusion:**

TMJT may alleviate DN via core components (e.g. Denudatin B, hancinol, hirudinoidine A) targeting SDECGs (e.g. SRC, EGF, GAPDH), with the involvement of feature genes and modulation of immune and inflammation-related pathways. These findings have potential implications for clinical practice and future investigations.

## 1 Introduction

Diabetic nephropathy (DN) is a significant public health challenge as it is the leading cause of chronic kidney disease and end-stage renal disease worldwide (1). It is a microvascular complication of diabetes mellitus (DM), and up to 40% of people with DM may develop DN (2, 3). DN is characterized by persistent albuminuria, a progressive decline in renal function, and an increased risk of cardiovascular disease and mortality (4, 5). The pathogenesis of DN involves multiple factors such as abnormal glucose metabolism, altered renal hemodynamics, oxidative stress, cytokine action, and genetic factors (6). Current therapies for DN focus on risk factor interventions in the early stages and renal replacement therapies in the end-stage, but they have limitations in preventing disease progression (7). Therefore, there is an urgent need to identify safe and effective treatments for DN.

Traditional Chinese medicine (TCM) is a promising approach for treating DN due to its safe and effective complementary and alternative therapies (8). Both TCM decoctions and Chinese Patent medicines have shown effectiveness in treating DN by improving kidney function, regulating immune function, and reducing urine protein and blood glucose levels (9, 10). TMJT is a Chinese patent medicine composed of ten herbs and one animal drug (**Table 1**) that has been used to treat DM and its neurological and vascular complications. Clinical studies (11, 12) and animal experiments (13) have demonstrated its significant therapeutic effect on diabetic peripheral neuropathy, improving clinical symptoms, nerve function, and tissue structure. Observational studies (14, 15) have also reported that TMJT can improve glucolipid metabolism and regulate the expression of inflammatory factors in the treatment of cerebral infarction (CI) combined with DM.

**Table 1:** Comparison of names of Chinese medicines in TMJT.

No.	Pinyin Name	Pharmaceutical Latin	English Name
1	Taizishen	*Radix Pseudostellariae*	Heterophylly falsestarwort root
2	Danshen	*Radix Salviae*	Red sage root
3	Huanglian	*Rhizoma Coptidis*	Chinese goldthread
4	Huangqi	*Radix Astragali*	Milkvetch root
5	Jiaogulan	*Herba Gynostemmae Pentaphylli*	Fiveleaf gynostemma herb
6	Shanyao	*Rhizoma Dioscoreae*	Chinese yam
7	Cangzhu	*Rhizoma Atractylodis*	Swordlike atractylodes rhizome
8	Xuanshen	*Radix Scrophulariae*	Figwort root
9	Shuizhi	*Hirudo*	Leech
10	Dongkuiguo	*Fructus Malvae*	Cluster mallow fruit
11	Gegen	*Radix Puerariae Lobatae*	Lobed kudzuvine root

**Figure 1:**
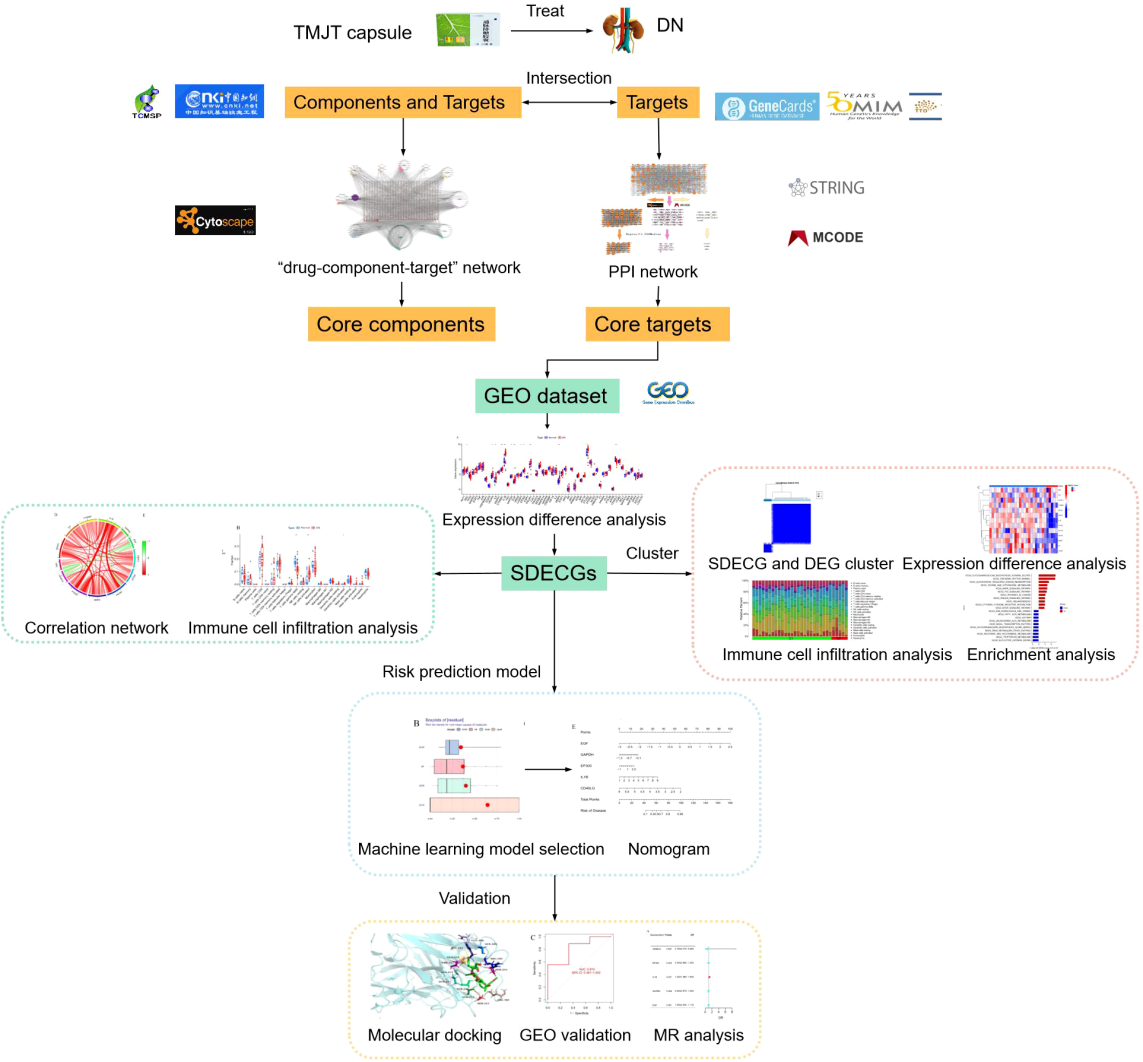
Flow diagram of this study.

TMJT has been reported to be widely used in the treatment of DN in previous studies. However, these studies have several limitations, such as being few in number, low in quality, and primarily conducted from a clinical or theoretical perspective, without focusing on mechanisms. Thus, the material basis of TMJT for DN and its molecular mechanisms require further exploration. Network pharmacology can analyze the patterns of molecular association between drugs and diseases from a systemic level and an overall perspective of biological networks, and is therefore widely used to discover active components and elucidate the overall mechanisms of TCM (16). Genome-wide transcriptome analysis, which utilizes microarray and bioinformatics technologies, can identify key targets for disease progression and provide insight into disease pathogenesis and molecular classification (17). MR is a method that uses single nucleotide polymorphisms (SNPs) as instrumental variables (IVs) to assess causal relationships between exposure factors and diseases (18). Therefore, in this study, network pharmacology was employed to analyze the core targets of TMJT in treating DN. Bioinformatics analysis was used to assess the expression of these core targets in the Gene Expression Omnibus (GEO) dataset. Additionally, MR analysis was conducted to investigate the causal relationship between core targets and DN, aiming to reveal their potential mechanisms of action. The process of this study can be seen in **Figure 1**.

## 2 Methods

### 2.1 Components and targets in TMJT

The components in TMJT were collected by retrieving the traditional Chinese medicine system pharmacology technology platform (TCMSP, http://tcmspw.com/tcmsp.php) (19) and the China national knowledge infrastructure (CNKI) reviews (The search term were the Chinese name of each Chinese medicine, and the search time was from January 1, 2018 to December 31, 2022). For the components available in the TCMSP database, filter conditions of oral bioavailability (OB) ≥ 30% and drug-likeness (DL) ≥ 0.18 were applied. For the components not included in the TCMSP database, the SwissADME (http://www.swissadme.ch/) (20) database was used for screening, with filter conditions of ‘high’ oral bioavailability and at least three ‘yes’ items in drug-likeness. Relevant targets were matched for components available in the TCMSP or the SwissTargetPrediction (http://www.swisstargetprediction.ch/) (21) was used to predict targets for the components not available in the TCMSP. The Universal Protein (Uniprot, http://uniprot.org/) (22) database was used for target matching.

### 2.2 Diabetic nephropathy-related targets

This study searched the Genecards (https://www.genecards.org/) (23), Online Mendelian Inheritance in Man (OMIM, https://omim.org/#) (24), and the Therapeutic Target Database (TTD, http://db.idrblab.net/ttd/) (25) for “diabetic nephropathy” to retrieve DN-related targets. The obtained targets were merged, and duplicates were removed.

### 2.3 Construction of “drug-component-target” network

The intersection of the relevant targets for each active component in TMJT and the DN-related targets was obtained using Excel software. Then, Cytoscape 3.8.0 was used to construct a network, taking disease, drugs, components, and relevant targets as nodes, and their mutual relationship as edges. A topological analysis was performed to identify the core components in the network.

### 2.4 Construction of protein-protein interaction network

The PPI network of the intersection targets was obtained from the String database (https://string-db.org) (26) by setting the species to Homo sapiens and the minimum required interaction score to 0.40. The core PPI targets were obtained using the MCODE plugin, which clusters the PPI network.

### 2.5 Collection and procession of GEO samples

We used “diabetic nephropathy” as a keyword and restricted the data type (Expression profiling by array) and organism (Homo sapiens) to retrieve samples from the GEO (https://www.ncbi.nlm.nih.gov/geo/) database. Gene expression and clinical data were obtained, and Perl code was used for gene symbol annotation and data correction to obtain the expression levels of the core genes of TMJT capsule for DN treatment obtained from network pharmacology in each sample of the normal and DN groups, respectively.

### 2.6 Expression difference of core genes, chromosome position, and expression correlation of significantly differently expressed core genes

The expression levels of the core genes were extracted from both the normal and DN groups, and differential expression analysis was performed using R packages such as “limma”, “pheatmap”, and “ggpubr”. The results were displayed using box plots and a heat map, with genes having a p-value < 0.05 defined as significantly differentially expressed core genes (SDECGs). The perl code was utilized to locate the core genes on the chromosomes, and R package “Rcircos” was used to represent them as circle plots. Furthermore, correlation coefficients for each SDECG were calculated using the “cor” command and visualized.

### 2.7 Infiltration, difference, and correlation of immune cells in DN samples

The relative content of immune cells, totaling 1, was obtained through 1,000 simulations using the CIBERSORT command in R. The content of immune cells in each sample was then visualized using a bar plot. Single-sample gene set enrichment analysis (ssGSEA) was performed using the R packages “GSVA” and “GSABase” to compare the differences in immune cell content between the normal and DN groups. The ssGSEA results were presented as box plots. The SDECGs were intersected with the ssGSEA scores, and correlation tests were performed to obtain correlation coefficients, which were then visualized.

### 2.8 Selection of machine learning model and nomogram for TMJT in treatment of DN

The expression data of SDECGs were utilized to construct four prediction models, namely random forest (RF), support vector machine (SVM), generalized linear (GL) and extreme gradient boosting (XGB) models. We defined the prediction functions and calculated the results of each of the four models. To screen feature genes in SDECGs, we created reverse cumulative distribution plot of residual, residual box plot, and receiver operating characteristic (ROC) curves for comprehensive consideration. After selecting the best model, we constructed a nomogram using the feature genes and their expression levels in the normal and DN groups. Finally, decision curve and calibration curve were constructed to assess the accuracy of the nomogram.

### 2.9 Molecular docking and GEO model validation

The 3D structures of the previously obtained core components of TMJT capsule and feature genes were obtained from Pubchem (https://pubchem.ncbi.nlm.nih.gov) (27) and Protein Data Bank (PDB) (http://www.rcsb.org/) (28) databases. Autodock Vina was used for molecular docking to preliminarily validate the interaction of core network pharmacological components with feature genes, and the best four combinations of docking were selected and visualized using Pymol. Another dataset containing normal and DN groups was acquired from the GEO database, and a machine learning model was constructed using the same method as before in the R language. ROC was plotted to validate the constructed machine learning model in 2.8.

### 2.10 MR analysis between feature genes and DN

We conducted two-sample MR analysis to explore the causal relationships between feature genes and the risk of DN, defining SNPs as IVs. We obtained SNPs of feature genes as exposure factors and SNPs of DN as outcome factors from the integrated epidemiology unit (IEU) database (https://gwas.mrcieu.ac.uk/). MR analysis was performed using the ‘TwoSampleMR’ package, and the relationship between feature gene expression levels and DN risk was assessed using the inverse variance weighted (IVW) method. We also employed Cochran’s Q statistic to test for heterogeneity, where p < 0.05 indicated heterogeneity in IVW results (29). Potential horizontal pleiotropy was evaluated using MR-Egger regression and MR-PRESSO analysis, with p < 0.05 indicating horizontal pleiotropy in IVW results (30, 31).

### 2.11 Clusters of SDECGs and analysis between SDECG clusters

We used the R package “ConsensusClusterPlus” to cluster DN samples according to the expression of SDRCG with a k-means clustering method, Euclidean distance type, and maximum of nine clusters. The resulting clusters were analyzed by comparing their expression levels using heat maps and box plots. Principal components analysis was also performed to assess the differentiation among clusters. An ssGSEA analysis of the SDECG clusters was then conducted to obtain a bar plot of the individual immune cell content of each sample in the different clusters and to compare the differences among the content of the immune cells in the different clusters. Gene ontology (GO) and Kyoto encyclopedia of genes and genomes (KEGG) enrichment analysis were performed using gmt files downloaded from the GSEA platform (http://www.gsea-msigdb.org/) and Gene set variation analysis (GSVA) was conducted using the R language to analyze the expression of the enrichment items among clusters. Finally, difference analysis was performed on gene expression of SDECG clusters with filtering conditions of |logFC| > 1 and adj. P-Value < 0.05, and the differentially expressed genes (DEGs) were obtained by taking the intersection of SDECG clusters using a Venn diagram.

### 2.12 Enrichment analysis in DEGs between SDECG clusters

The DEGs between the SDECG clusters were subjected to biological process (BP), molecular function (MF), and cellular component (CC) gene ontology (GO) enrichment analysis, as well as KEGG pathway enrichment analysis. These analyses were conducted using R packages such as “clusterProfiler” and “enrichplot”, with a screening condition of p-value < 0.05. The results were visualized as circles plots and bar plots.

### 2.13 Clusters of DEGs and analysis among DEG clusters

We conducted another cluster analysis according to the expression of DEGs using the same clustering method as in 2.11, and the DEG cluster with the highest accuracy was selected. The expression levels of DEGs in different clusters, the differences in SDECG expression, and immune cell content among different clusters were compared based on the DEG clustering results. These results were visualized using a heat map and box plots, respectively.

### 2.14 SDECG scores and differential analysis, and construction of alluvial plot

We utilized the PCA method to calculate the SDECG scores for each sample by summing up PC1 and PC2 based on the expression levels of SDECGs (32). Differential analysis was performed on the SDECG scores of both the SDECG clusters and DEG clusters using R packages such as “limma” and “ggpubr”. Box plots were created to illustrate the SDECG scores of samples clustered in SDECGs and DEGs. Additionally, an alluvial diagram was drawn using the R package “ggalluvial” to visualize the relationships and overall processes among the SDECG clusters, DEG clusters, and samples with high and low SDECG scores.

### 2.15 Statistical analysis

In network pharmacology, we utilized Cytoscape V3.8.0 and its plugins for network analysis. The topological analysis of the “drug-component-target” network was mainly based on the degree of nodes. For PPI network analysis, we applied the MCODE plugin of Cytoscape with degree cut-off = 2, node score cut-off = 0.2, K-core = 2, and max. depth = 100 to identify clusters in the PPI network. Core targets were also screened out based on their node degree. Molecular docking was performed with an energy range of 5, exhaustiveness of 400, and 20 models to obtain binding energy combinations. For the extraction of GEO files and data annotation, we utilized Strawberry Perl 5.32.1.1, while R V4.1.2 was used for all statistical analyses. In this study, t-tests were used for the comparison of two independent samples, while the Wilcoxon paired rank sum test was utilized for two paired samples. For data with three or more groups, we used one-way analysis of variance (ANOVA) and Kruskal-Wallis rank sum test, while the Spearman rank correlation test was used for correlation analysis. Statistical significance was set at P-value < 0.05 or false discovery rate (FDR) (Benjamini-Hochberg method) corrected for P-value < 0.05. In MR analysis, we established the following criteria: 1) SNPs selected should exhibit a strong correlation with the exposure factor, with corresponding p-values < 5 × 10^-8; 2) During the process of linkage disequilibrium clumping, we set the r² threshold at 0.001; 3) For the clumping analysis, we defined a window size of 10,000 kilobase pairs (33); 4) The F-statistic for the SNPs associated with the exposure factor should be > 10 (34); 5) We applied Bonferroni correction to adjust the threshold for significance level (35).

## 3 Results

### 3.1 Collection of components and targets in TMJT

We searched the TCMSP database and CNKI reviews (36–39), and combined the results with SwissADME and SwissTargetPrediction to collect information on active components and related targets of TMJT. After removing duplicates and excluding irrelevant data, we obtained 184 unique active components, identified ten repeated components, and 515 associated targets. Detailed information on these components is available in **Supplementary Table S1**.

### 3.2 Collection of DN related targets

After obtaining 1,278, 68, and 22 targets from Genecards, OMIM, and TTD respectively, we obtained 1,063 DN-related targets after processing. We then used Excel to perform an intersection between the drug and disease targets, resulting in 229 targets directly related to both drugs and diseases.

### 3.3 Analysis of “drug-component-target” network

**Figure 2:**
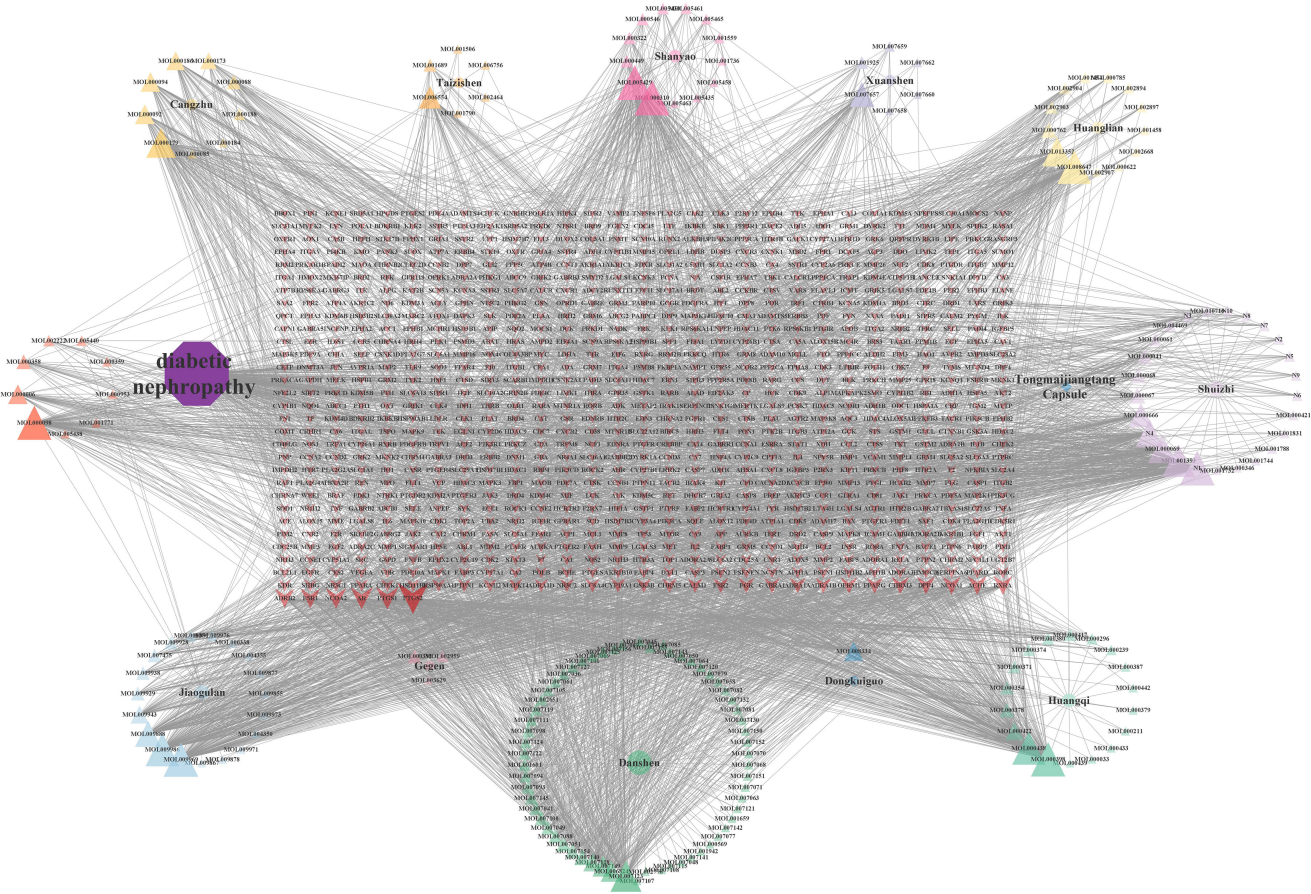
Drug-component-target network. There were 12 kinds of herbs, 184 compounds, and 1,106 related targets on the network. The purple octagon represents DN. The blue diamond represents TMJT. Eleven circles represent eleven kinds of Chinese medicines, each color represents one medicine. Triangles represent the components of different Chinese medicines, and the components have the same color as their source of Chinese medicines. The red arrow represents the target of the intersection of drug and disease. The transparency of the node reflects the degree value of the node.

The “drug-component-target” network was constructed using Cytoscape V3.8.0 and its plugins, with 1,073 nodes and 4,029 edges, as illustrated in **Figure 2**. Seventeen core active components, including Denudatin B, hancinol, hirudinoidine A, Gypenoside XXXV_qt, isoflavanone, quercetin, Moupinamide, Gypentonoside A_qt, (3R)-3-(2-hydroxy-3,4-dimethoxyphenyl)chroman-7-ol, Myristic acid, miltirone II, bacunone,2-Hydroxyisoxypropyl-3-hydroxy-7-isopentene-2,3-dihydrobenzofuran-5-carboxylic, palmitic acid, Methyl 4-methyltetradecanoate, Gypenoside XXXVI_qt, and scropolioside A_qt, were identified based on their node degree ≥ 60. These core components are considered to be the main material basis for TMJT to treat DN.

### 3.4 Analysis of PPI network

**Figure 3:**
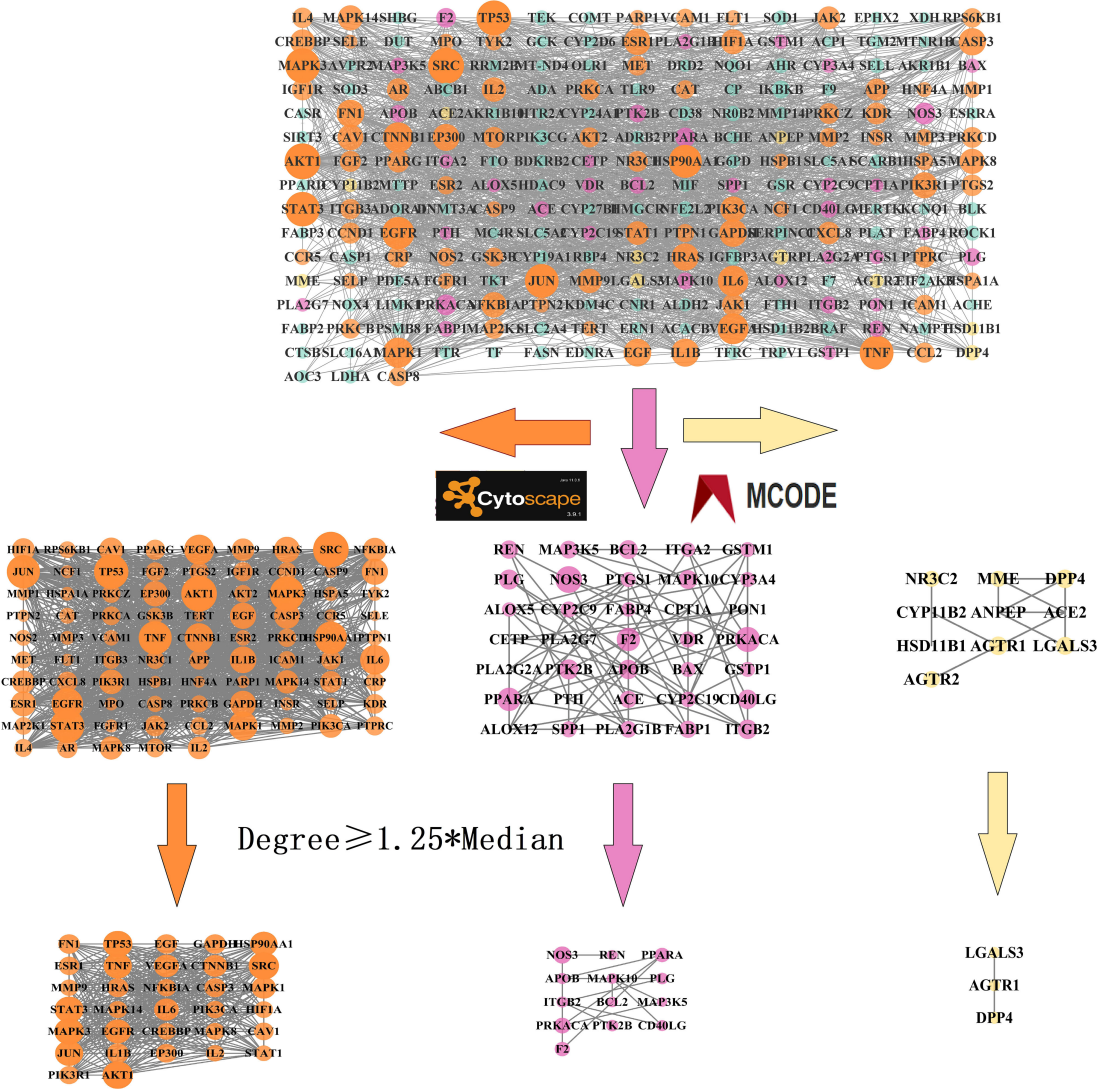
PPI network of intersection targets. The nodes in the network represent the intersection targets of TMJT and DN. The orange, pink and yellow nodes represent cluster 1, cluster 2 and cluster 3 respectively. The size of each node reflects the degree value.

We constructed a PPI network using the intersection targets obtained from STRING and Cytoscape, with 229 nodes and 4,154 edges, and an average node degree of 37.4. The PPI network showed significant clustering with a p-value less than 1.0e-16. We used the MCODE plugin to cluster the PPI network and obtained three clustering networks, as depicted in **Figure 3**. To identify the core targets, we selected targets with a node degree greater than or equal to 1.25 times the median in each clustering network. We identified 48 core targets, which are considered to be the main targets of TMJT for treating DN, including *AKT1*, *TNF*, *EGFR*, *STAT3*, *SRC*, *IL6*, *NOS3*, *PPARA*, and *AGTR1*. Further information on the core targets is provided in **Table 2**.

**Table 2:** Information of core targets.

Gene symbol	Gene name	Cluster	Degree
*FN1*	Fibronectin	1	46
*TP53*	Cellular tumor antigen p53	1	66
*EGF*	Pro-epidermal growth factor	1	46
*GAPDH*	Glyceraldehyde-3-phosphate dehydrogenase	1	46
*HSP90AA1*	Heat shock protein HSP 90-alpha	1	61
*ESR1*	Estrogen receptor	1	43
*TNF*	Tumor necrosis factor	1	63
*VEGFA*	Vascular endothelial growth factor A	1	55
*CTNNB1*	Catenin beta-1	1	53
*SRC*	Proto-oncogene tyrosine-protein kinase Src	1	69
*MMP9*	Matrix metalloproteinase-9	1	37
*HRAS*	GTPase HRas	1	45
*NFKBIA*	NF-kappa-B inhibitor alpha	1	40
*CASP3*	Caspase-3	1	45
*MAPK1*	Mitogen-activated protein kinase 1	1	53
*STAT3*	Signal transducer and activator of transcription 3	1	65
*MAPK14*	Mitogen-activated protein kinase 14	1	39
*IL6*	Interleukin-6	1	54
*PIK3CA*	Phosphatidylinositol 4,5-bisphosphate 3-kinase catalytic subunit alpha isoform	1	40
*HIF1A*	Hypoxia-inducible factor 1-alpha	1	38
*MAPK3*	Mitogen-activated protein kinase	1	66
*EGFR*	Epidermal growth factor receptor	1	59
*CREBBP*	CREB-binding protein	1	38
*MAPK8*	Mitogen-activated protein kinase 8	1	39
*CAV1*	Caveolin-1	1	41
*JUN*	Transcription factor Jun	1	63
*IL1B*	Interleukin-1 beta	1	47
*EP300*	Histone acetyltransferase p300	1	44
*IL2*	Interleukin-2	1	37
*STAT1*	Signal transducer and activator of transcription 1-alpha/beta	1	35
*PIK3R1*	Phosphatidylinositol 3-kinase regulatory subunit alpha	1	42
*AKT1*	RAC-alpha serine/threonine-protein kinase	1	69
*NOS3*	Nitric oxide synthase, endothelial	2	30
*REN*	Renin	2	15
*PPARA*	Peroxisome proliferator-activated receptor alpha	2	24
*APOB*	Apolipoprotein B-100	2	17
*MAPK10*	Mitogen-activated protein kinase 10	2	16
*PLG*	Plasminogen	2	18
*ITGB2*	Integrin beta-2	2	17
*BCL2*	Apoptosis regulator Bcl-2	2	16
*MAP3K5*	Mitogen-activated protein kinase kinase kinase 5	2	14
*PRKACA*	cAMP-dependent protein kinase catalytic subunit alpha	2	27
*PTK2B*	Protein-tyrosine kinase 2-beta	2	18
*CD40LG*	CD40 ligand	2	17
*F2*	Prothrombin	2	24
*LGALS3*	Galectin-3	3	11
*AGTR1*	Type-1 angiotensin II receptor	3	12
*DPP4*	Dipeptidyl peptidase 4	3	12

### 3.5 Acquisition of samples in GEO datasets

By searching the GEO database for “diabetic nephropathy” and limiting the data type and species, we selected two datasets: dataset GSE142153 containing ten normal samples and 30 DN samples, and dataset GSE30122 containing 50 normal samples and 19 DN samples. The former was used for analysis and model construction, while the latter was used to validate the analysis results.

### 3.6 Analysis of core gene expression difference, chromosome position, and expression correlation of SDECGs

**Figure 4:**
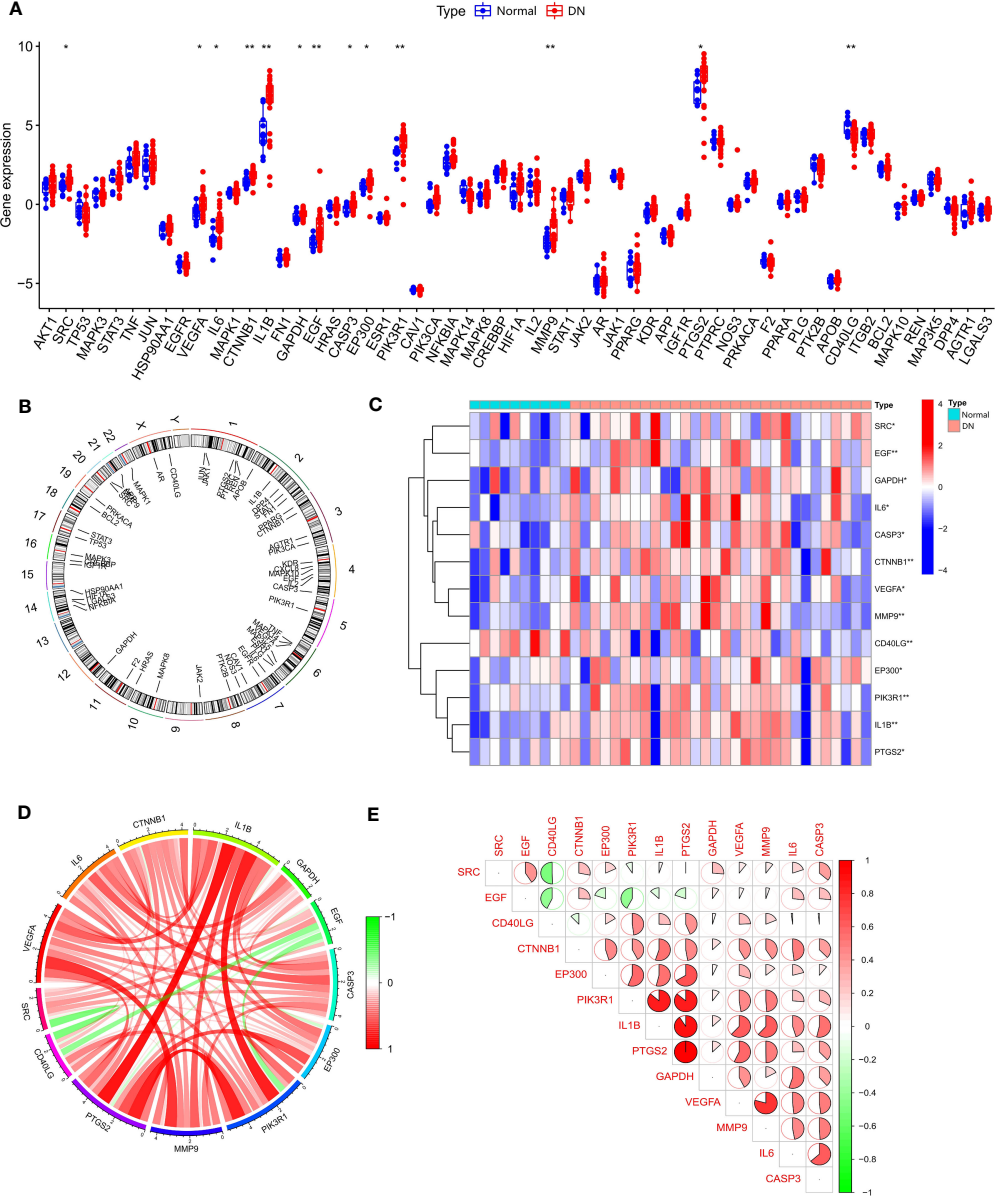
**(A)** Box plot of expression difference analysis of core genes between normal samples and DN samples; **(B)** Circle plot of chromosome location of core genes; **(C)** Heat map of SDECG expression in normal and DN samples; **(D)** SDECG correlation network; **(E)** Correlation analysis between the two SDECGs. * p<0.05; ** p<0.01.

We obtained 48 TMJT core genes through network pharmacology analysis, as shown in **Table 2**. Differential analysis between the DN group and the normal group of dataset GSE142153 revealed that 13 genes, including *SRC*, *EGF*, *GAPDH*, *IL6*, *CASP3*, *CTNNB1*, *VEGFA*, *MMP9*, *CD40LG*, *EP300*, *PIK3R1*, *IL1B*, and *PTGS2*, were SDECGs which are the core genes that have been screened out after validation by human samples, thus they have more accuracy and clinical value. Among these genes, all were highly expressed in the DN group, except for CD40LG, which was highly expressed in the normal group, as demonstrated in **Figures 4A, B**. The specific chromosomal locations of the TMJT core genes can be found in **Figure 4C**. Correlation analysis between every two SDECGs in DN samples indicated a strong correlation between the SDECGs, with the correlation being primarily positive, as displayed in **Figures 4D, E**.

### 3.7 Analysis of normal and DN samples in immune cell infiltration, difference, and correlation

**Figure 5:**
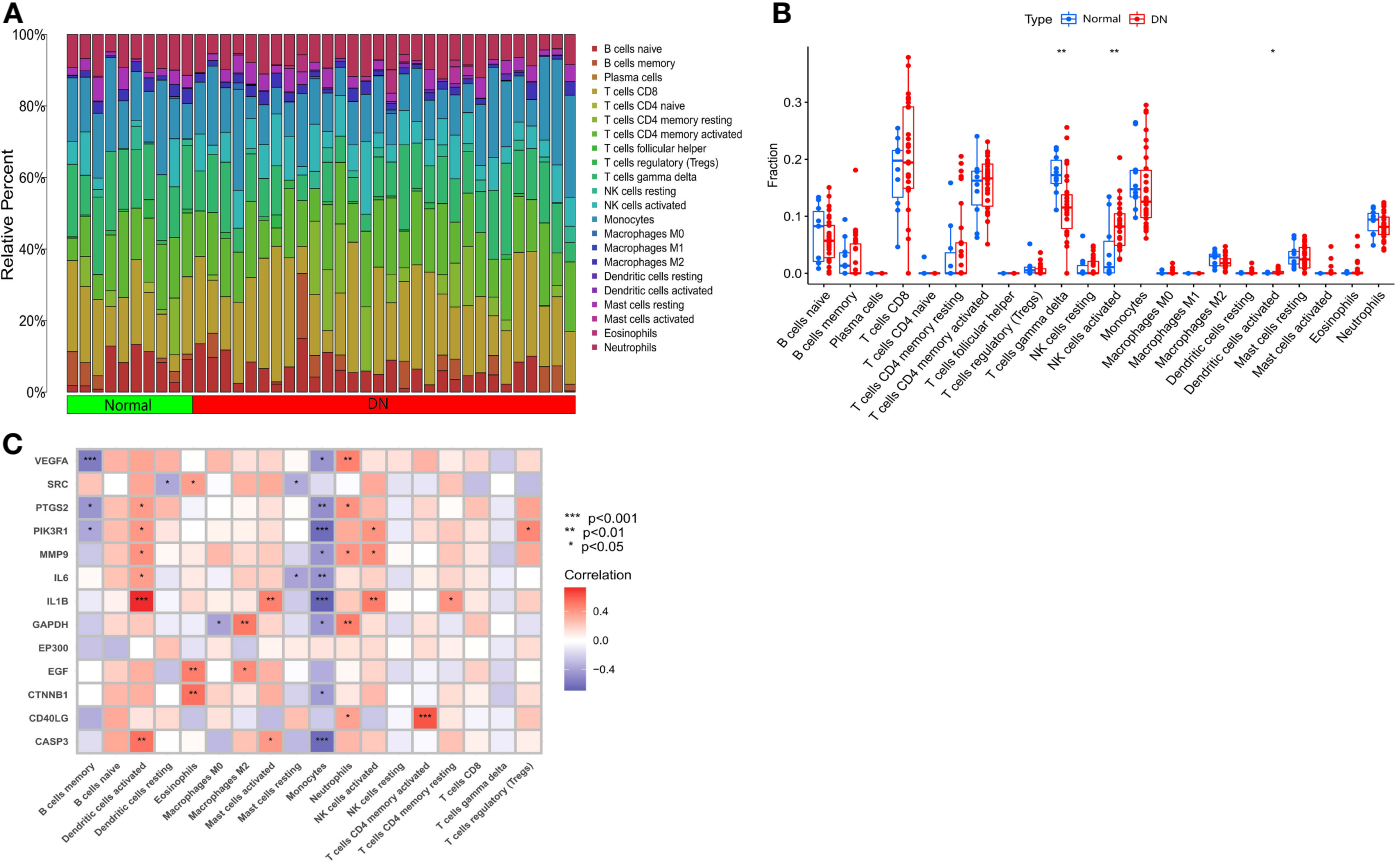
**(A)** Bar plot of relative percentage of each immune cells in samples; **(B)** Box plot of immune cell fraction between normal samples and DN samples; **(C)** Heat map of correlation analysis between SDECGs and immune cells. * p<0.05; ** p<0.01; *** p<0.001.

To explore the mechanisms between the DN group and the normal group from different levels, we conducted an immune cell infiltration analysis to determine the type and content of immune cells expressed in each sample, and the results are presented in **Figure 5A**. We also performed ssGSEA (**Figure 5B**) to identify statistically significant immune cells in the normal and DN groups, which included T cells gamma delta (with high expression in the normal group), NK cells activated (with high expression in the DN group), and activated Dendritic cells (with high expression in the DN group). Immune cell correlation analysis (**Figure 5C**) revealed that some correlations were primarily positive between SDECGs and immune cells. Among the immune cells with significant correlations (P < 0.05) with SDECGs, B cells memory, Monocytes, and Dendritic cells resting were mainly negatively correlated with related SDECGs, while activated Dendritic cells, Eosinophils, Macrophages M2, Mast cells activated, Neutrophils, activated NK cells, activated T cells CD4 memory, resting T cells CD4 memory, and T cells regulatory were mainly positively correlated with related SDECGs.

### 3.8 Selection of machine learning models and construction of nomogram of DN probability

**Figure 6:**
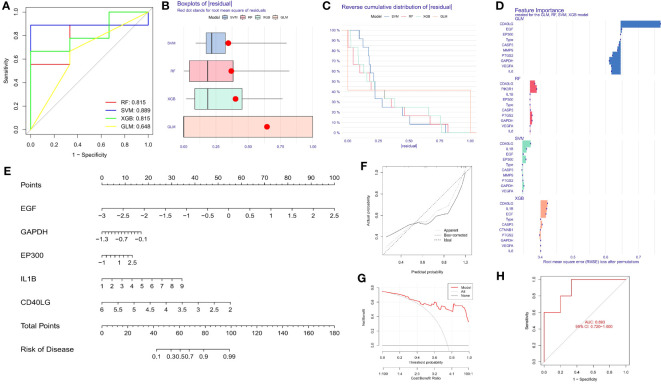
**(A)** ROC of the four machine learning models; **(B)** Box plots of residual of the four machine learning models; **(C)** Reverse cumulative distribution of residual of the four machine learning models; **(D)** Bar plot of feature importance of the four machine learning models; **(E)** Nomogram of the feature genes; **(F)** Calibration curve of feature genes nomogram of TMJT in treating DN; **(G)** Decision curve of feature genes nomogram of TMJT in treating DN; **(H)** ROC of the test GEO dataset.

We utilized the data of SDECGs to construct four machine learning prediction models: SVM, RF, XGB, and GLM. ROC curves, residual box plots, and reverse cumulative distribution plots were analyzed, revealing that the SVM method had the highest accuracy, with the largest area under the ROC (AUC) and the lowest residuals and reverse cumulative values (**Figures 6A–C**). Therefore, we selected SVM as the best model for further construction. The SVM model was then used to obtain the importance scores of feature genes, as illustrated in **Figure 6D**, which revealed nine feature genes in order of importance scores: *CD40LG*, *EP300*, *IL1B*, *GAPDH*, *EGF*, *PTGS2*, *MMP2*, *CASP3*, and *VEGFA*. *CD40LG* had the highest importance score among them. We used the top five genes (*CD40LG*, *EP300*, *IL1B*, *GAPDH*, *EGF*) to construct a nomogram, where we obtained individual score scales for these genes (**Figure 6E**). Treatment sensitivity was determined by calculating the sum of the expression scores of these feature genes to predict the risk rate of TMJT treatment of DN feature genes in the development of DN. The accuracy of this prediction was high, as evidenced by the close proximity of the solid and dashed lines in the calibration curve (**Figure 6F**), the distance between the red and gray lines in the decision curve (**Figure 6G**), and the AUC of 0.893 (>0.720 [95% CI]) in the ROC curve (**Figure 6H**).

### 3.9 Molecular docking validation and GEO datasets validation

**Figure 7:**
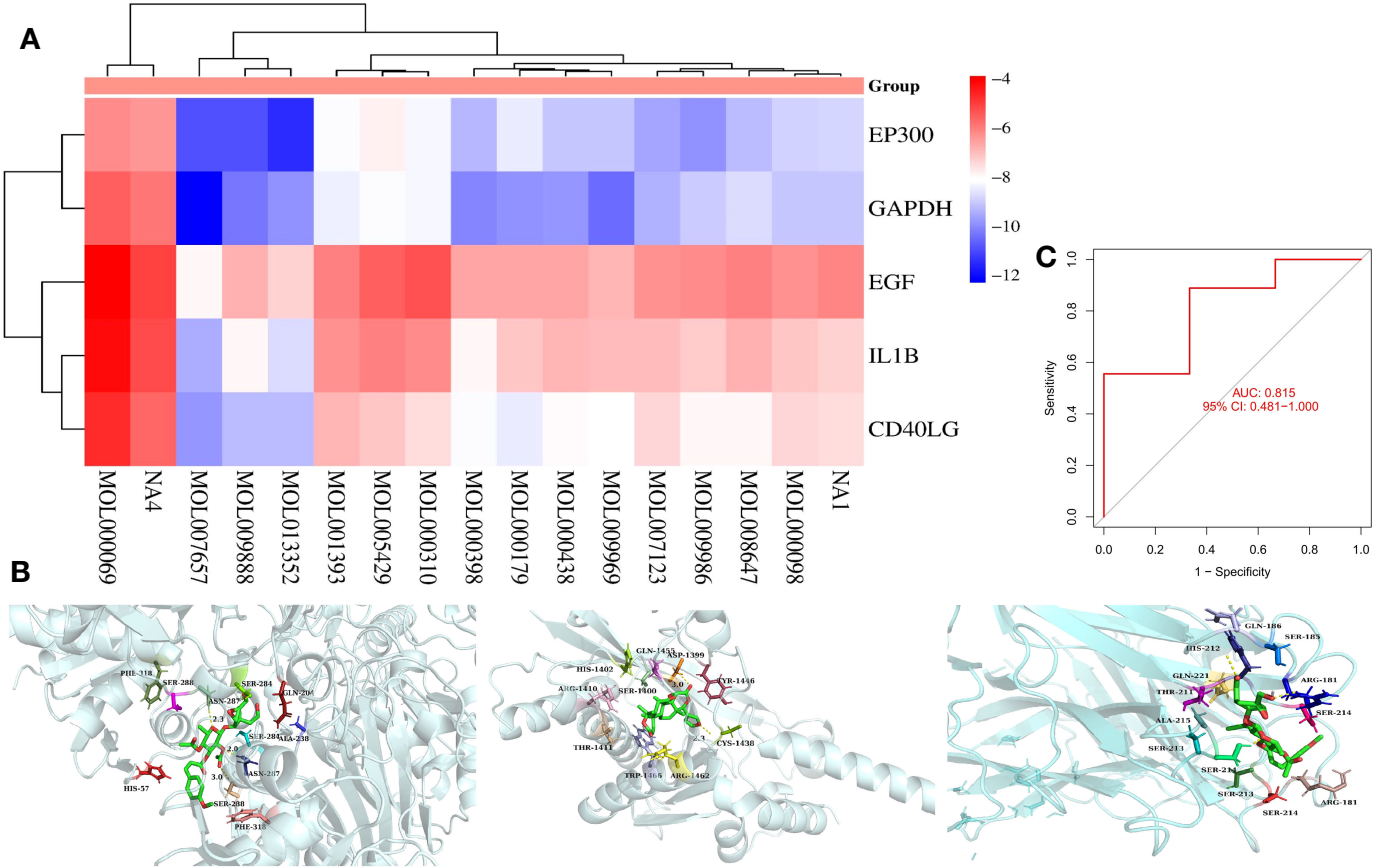
**(A)** Heat map of molecular docking results between core components and feature genes; **(B)** The docking models of the best combinations (scropolioside A_qt and GAPDH, obacunone and EP300, gypenoside XXXVI_qt, and EP300); **(C)** ROC for model validation of GEO dataset construction.

To verify whether the core components of TMJT and the proteins encoded by the feature genes of TMJT for treating DN with the most clinical value that we obtained can bind and exert effects, we performed molecular docking on them. We found that the majority of docking combinations had binding energy lower than -5.0 kcal/mol through molecular docking analysis of the feature genes and core components of TMJT. This suggests that stable structures could form between most of the feature genes and core components. See **Figure 7A**; **Supplementary Table S2** for details, including binding energy, and the best docking combinations are shown in **Figure 7B**. Notably, scropolioside A_qt showed a strong binding affinity with residues GLN-204, ALA-238, SER-284, ASN-287, SER-288, and PHE-318 of GAPDH through hydrogen bonding, with a docking energy of -12.3 kcal/mol. Additionally, Obacunone interacted with EP300 through hydrogen bonding, involving ASP-1399, SER-1400, HIS-1402, ARG-1410, THR-1411, CYS-1438, TYR-1446, GLN-1455, ARG-1462, and TRP-1466, with a docking energy of -11.5 kcal/mol. Moreover, scropolioside A_qt was found to bind to residues ARG-181, SER-185, GLN-186, HIS-212, SER-213, SER-214, ALA-215, and GLN-221 of CD40LG through hydrogen bonding, with a docking energy of -9.8 kcal/mol.

We used the SVM method to construct a model for the expression data sample genes in the validation dataset (GSE30122) of DN, and brought the top five feature genes with feature importance scores into the model for validation. We plotted the ROC of the model (**Figure 7C**) and found that the AUC value was 0.815, which is greater than 0.481 [95% CI], indicating that the model with the GEO dataset has a high accuracy upon validation.

### 3.10 Results of MR analysis between feature genes and DN

**Figure 8:**
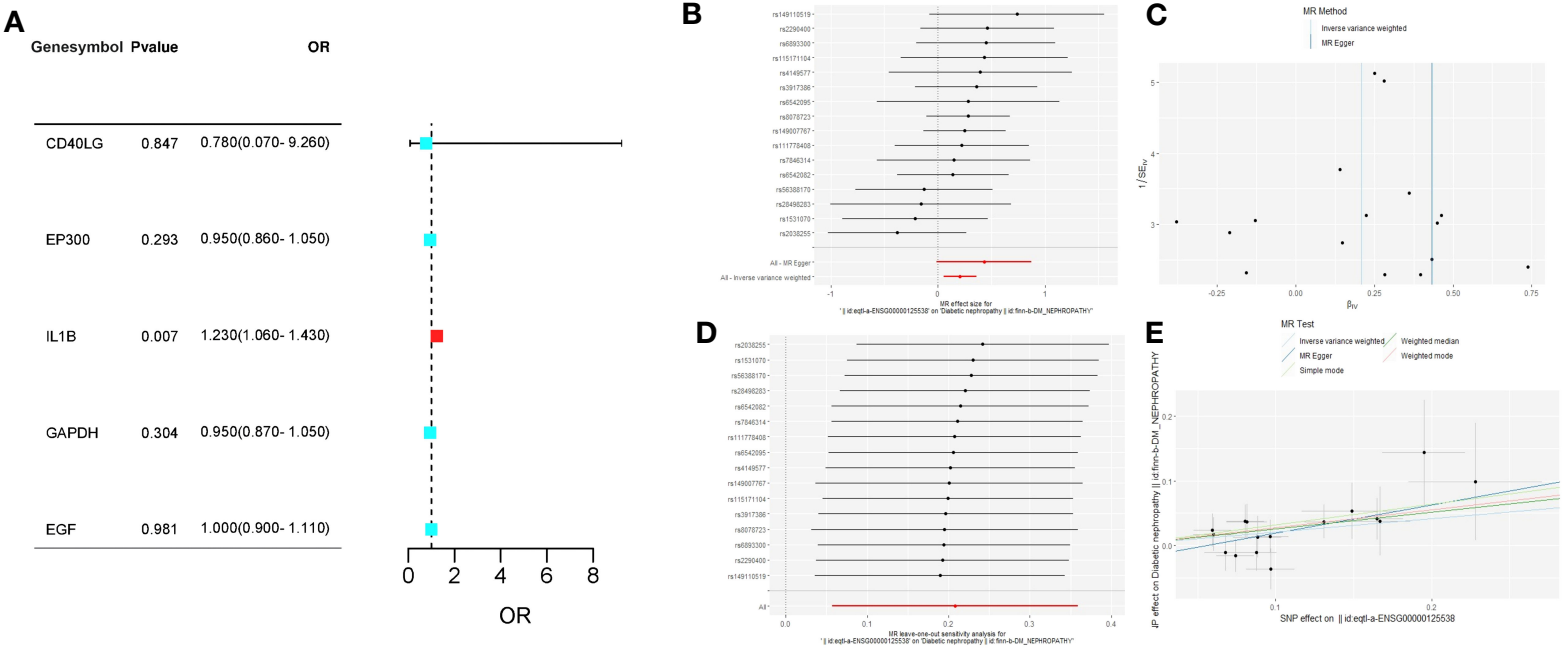
**(A)** The forest plot of the causal relationships between the five feature genes and DN under the IVW method; **(B)** The forest plot of the causal effects of each SNP in IL1B on the risk of DN; **(C)** Funnel plot of IL1B on DN; **(D)** Leave-one-out plot of IL1B on DN risk when leaving one SNP out; **(E)** Scatter plot of the causal effect of IL1B on the risk of DN.

We chose MR analysis to analyze the relationship between the feature genes and DN to verify whether their relationship was causal or correlational. Information on SNPs of the five feature genes (*CD40LG*, *EP300*, *IL1B*, *GAPDH*, *EGF*) in section 3.8 can be found in **Supplementary Table S3**, and none of the SNPs are weak IVs. The causal effects of each feature gene on DN can be seen in **Figure 8A**. After Bonferroni correction (p=0.05/5), IVW analysis reveals that IL1B levels are associated with an increased risk of DN (OR, 1.23; 95%CI, 1.06-1.43; P=0.007), while there is no significant causal relationship between the other four feature genes and DN (**Figure 8A**; **Supplementary Table S4**). For the IVW analysis of IL1B, its causal impact on DN can be seen in **Figures 8B, E**. The funnel plot of causal effects is approximately symmetrical (**Figure 8C**). Leave-one-out analysis demonstrates that systematically conducting MR analysis on the remaining SNPs after removing each SNP produces consistent results (**Figure 8D**), indicating the robustness of this finding.

The Cochran’s Q test did not reveal any heterogeneity in the IL1B results (p > 0.05). MR-Egger regression and MR-PRESSO analysis indicated the absence of horizontal pleiotropy in the *IL1B* results (p > 0.05; **Supplementary Table S5**).

### 3.11 Clusters of SDECGs of DN samples and analysis between SDECG clusters

**Figure 9:**
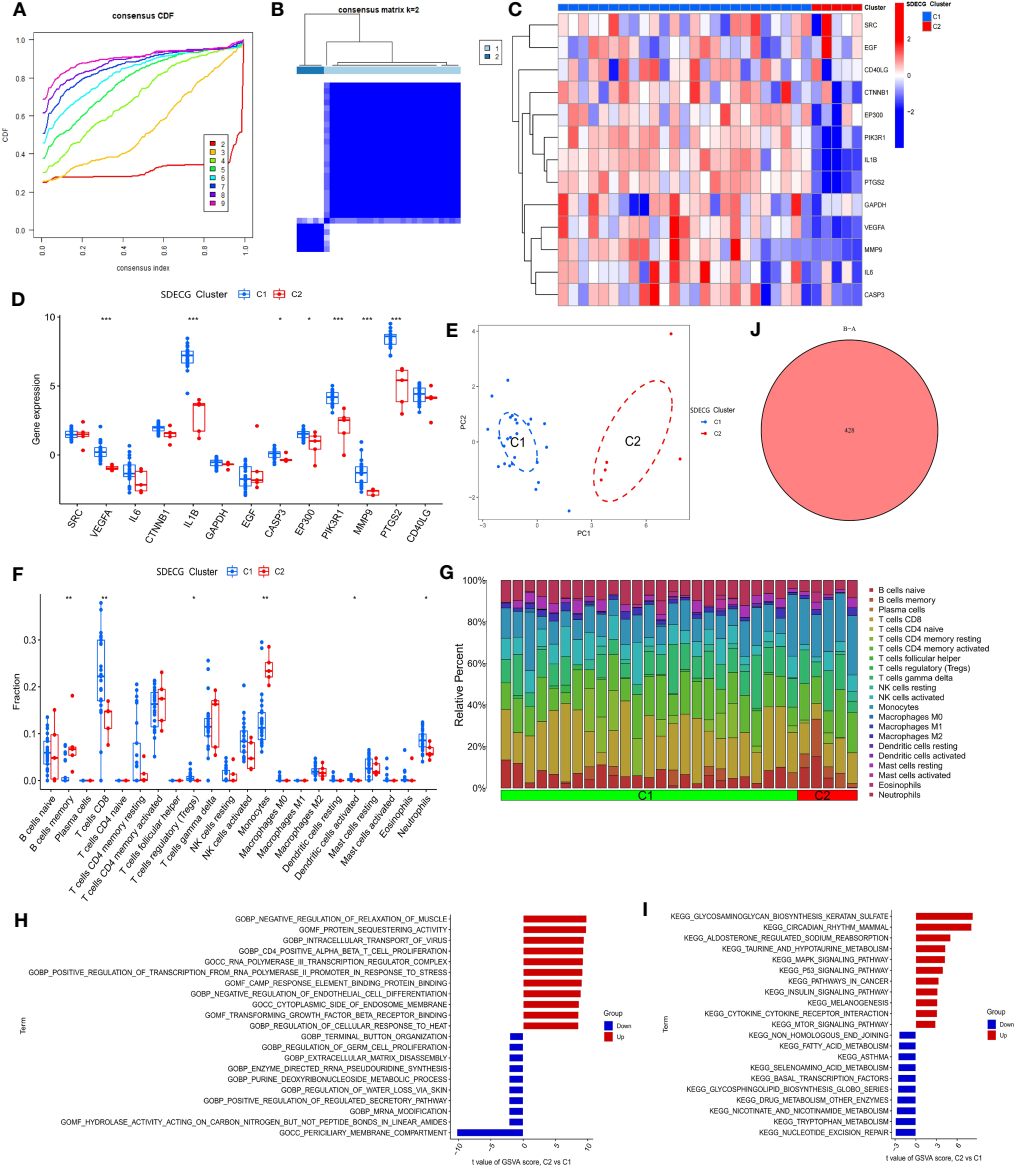
**(A)** Consensus cumulative distribution plot of SDECG clustering for samples; **(B)** Consensus matrix heat map of SDECG clustering for samples; **(C)** Heat map of SDECG expression between SDECG clusters; **(D)** Box plot of expression difference analysis of SDECG clusters; **(E)** Scatter plot of PCA between SDECG clusters; **(F)** Box plot of immune cell fraction between SDECG clusters; **(G)** Bar plot of relative percentage of each immune cells in samples of SDECG clusters; **(H)** Bar plot of GO terms of GSVA between SDECG clusters; **(I)** Bar plot of KEGG terms of GSVA between SDECG clusters; **(J)** Venn plot of DEGs. * p<0.05; ** p<0.01; *** p<0.001.

We clustered the samples based on the expression of SDECGs and found that the highest accuracy was achieved by dividing them into two clusters, as shown in **Figure 9A**. This resulted in the DN samples being divided into C1 and C2 groups, as illustrated in **Figure 9B**. By analyzing the inter-cluster differences, we can supplement and validate our previous results. We then analyzed the expression of SDECGs in the samples from both clusters, as depicted in **Figures 9C, D**. It was observed that all 13 SDECGs exhibited high expression in C1 and low expression in C2. Several genes, including *VEGFA*, *IL1B*, *CASP3, EP300, PIK3R1, MMP9, and PTGS2*, showed significant differences in expression between the two clusters. PCA (**Figure 9E**) revealed that SDECGs can distinguish between C1 and C2. Furthermore, ssGSEA analysis (**Figure 9F**) identified immune cells with significantly different expression between C1 and C2. B cells memory and Monocytes had down-regulated expression in C1, whereas T cells CD8, T cell regulatory, Dendritic cells activated, and Neutrophils had up-regulated expression in the C1 group. The distribution of different immune cells in each sample of C1 and C2 is shown in **Figure 9G**.

In the GSVA analysis, as shown in **Figures 9H, I**, it can be observed that compared to C1, C2 exhibits upregulation of GO BP terms related to negative regulation of muscle relaxation, intracellular transport of virus, CD4 positive alpha-beta T cell proliferation, among others. On the other hand, GO BP terms related to terminal button organization, regulation of germ cell proliferation, extracellular matrix disassembly, and others are mainly down-regulated in C2. GO MF terms such as protein sequestering activity, cAMP response element binding protein binding, and transforming growth factor beta receptor binding are mainly up-regulated in C2, while hydrolase activity acting on carbon-nitrogen but not peptide bonds in linear amides, and others are mainly down-regulated. Similarly, GO CC terms such as RNA polymerase III transcription regular complex and cytoplasmic side of endosome membrane are mainly up-regulated in C2, whereas the periciliary membrane compartment is mainly down-regulated. In terms of KEGG terms, glycosaminoglycan biosynthesis keratan sulfate, circadian rhythm mammal, and aldosterone-regulated sodium reabsorption, among others are mainly up-regulated in C2, while non-homologous end joining, fatty acid metabolism, asthma, and others are mainly down-regulated. Finally, genes with significant differences in expression between C1 and C2 samples were screened, resulting in a total of 428 DEGs (**Figure 9J**).

### 3.12 Enrichment analysis of DEGs between SDECG clusters

**Figure 10:**
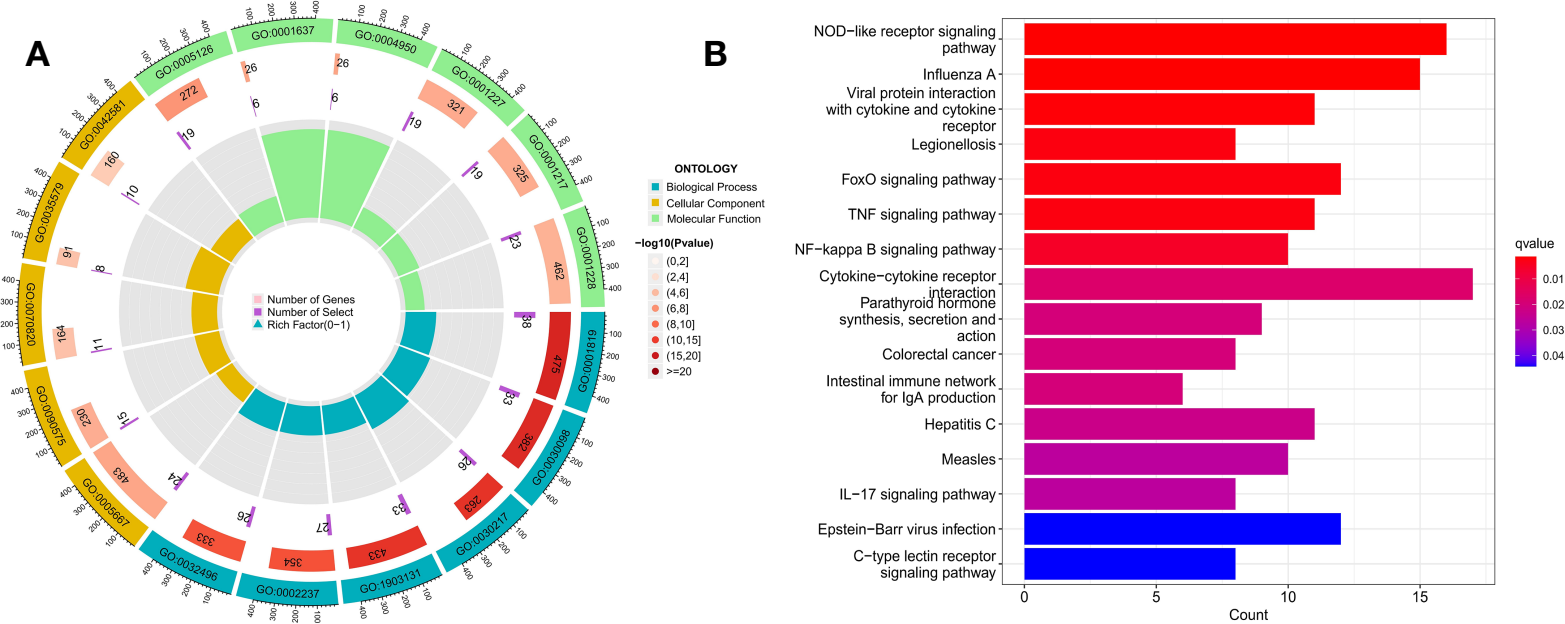
**(A)** GO enrichment analysis of DEGs; **(B)** KEGG pathway enrichment analysis of DEGs.

We performed enrichment analysis to obtain the biological functions and pathways of the 428 DEGs, thus obtaining the mechanisms of TMJT acting on DN from different dimensions to supplement our results. Among the results, GO analysis revealed that the significant biological processes (BPs) were mainly related to immune response, including positive regulation of cytokine production, lymphocyte differentiation, T cell differentiation, mononuclear cell differentiation, response to molecule of bacterial origin, and response to lipopolysaccharide, as shown in **Figure 10A**. In terms of cellular components, the DEGs were mainly involved in transcription regulation complexes, RNA polymerase II transcription regulator complexes, tertiary granules, specific granule membranes, and specific granules. The molecular functions of the DEGs were mainly related to cytokine receptor binding, G protein-coupled chemoattractant receptor activity, chemokine receptor activity, DNA-binding transcription repressor activity, RNA polymerase II-specific, DNA-binding transcription activator activity, and RNA polymerase II-specific. KEGG pathway enrichment analysis showed that the DEGs were mainly involved in NOD-like receptor signaling pathway, influenza A, viral protein interaction with cytokine and cytokine receptor, legionellosis, FoxO signaling pathway, and TNF signaling pathway, as shown in **Figure 10B**.

### 3.13 Clusters of DEGs and analysis between DEG clusters

**Figure 11:**
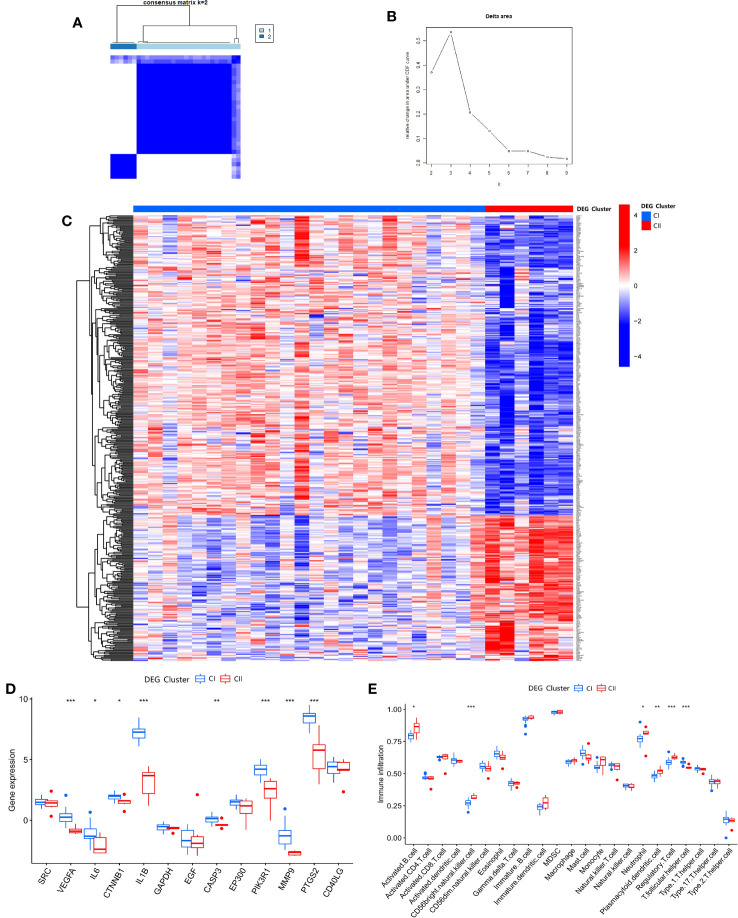
**(A)** Consensus matrix heat map of DEG clustering for samples; **(B)** Delta area plot of DEG clusters; **(C)** Heat map of DEG expression between DEG clusters; **(D)** Box plot of expression difference analysis of DEG clusters; **(E)** Box plot of immune cell infiltration between DEG clusters. * p<0.05; ** p<0.01; *** p<0.001.

After clustering the 428 DEGs from the SDECG clusters and selecting the best-clustered results, two clusters, namely CI and CII, were obtained (**Figures 11A, B**). Expression analysis of these two clusters’ samples showed that 285 DEGs, such as *CHRNA10*, *C5AR1*, *GPRIN3*, *IGSF22*, and *PDE2A*, were mainly highly expressed in CI and lowly expressed in CII, while 143 differential genes, such as CXCL10, OAS1, OAS2, IFIH1, and SAMD9L, were highly expressed in CI and lowly expressed in CII (**Figure 11C**).

Differential analysis of SDECGs for samples clustered by DEGs, as shown in **Figure 11D**, shows that these genes are up-regulated for CI expression and down-regulated for CII expression, among which the genes with significant differences are *VEGFA*, *IL6*, *CTNNB1*, *IL1B*, *CASP3*, *PIK3R1*, *MMP9*, *PTGS2*. ssGSEA of DEGs clustering (using the same gene set file as above) (**Figure 11E**) yielded the following immune cells with statistically significant differences: activated B cell, CD56 bright natural killer cell, neutrophil, plasmacytoid dendritic cell, regulatory T cell, and T follicular helper cell.

### 3.14 Scores of SDECGs, differential analysis of SDECG scores among clusters, and construction of alluvial plot

**Figure 12:**
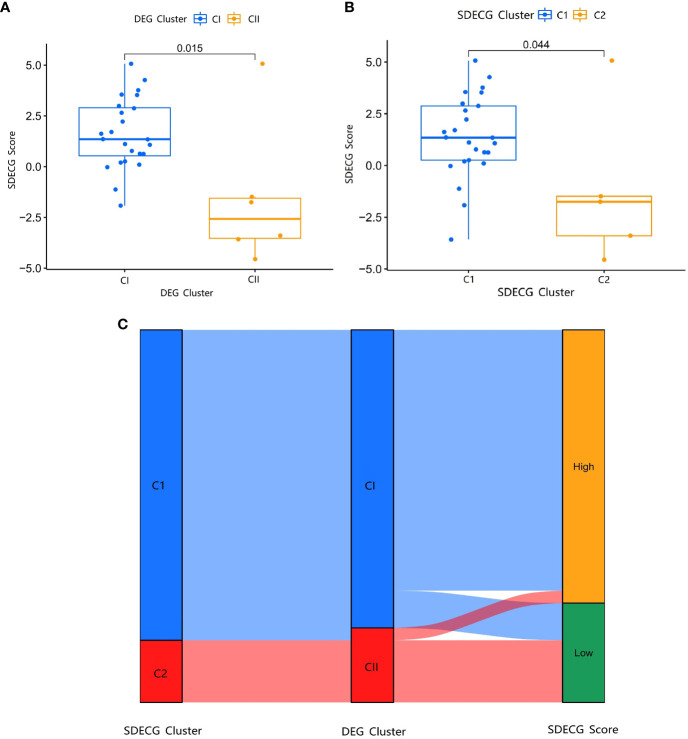
**(A)** Box plot of different expression analysis of SDECG score between DEG clusters; **(B)** Box plot of different expression analysis of SDECG score between SDECG clusters; **(C)** Alluvial plot of the correspondence of the different clustered samples.

We used PCA to analyze the SDECGs of TMJT for treating DN and built a scoring model. We compared the score differences between different clusters to determine whether the SDECGs had significant differences between them, and to identify the corresponding relationship of the clusters, ensuring the robustness of our results. The difference analysis of the SDECG clusters scored by the PCA method revealed a statistically significant difference between the two clusters (**Figures 12A, B**). Cluster 1 showed high scores, while Cluster 2 showed low scores. Similarly, a statistical difference was observed between the two clusters of DEGs scored by two clusters, with CI high and CII low. The alluvial plot in **Figure 12C** indicates that C1 of SDECG clustering primarily corresponds to CI of DEG clustering, while C2 of the former mainly corresponds to CII of the latter. Furthermore, high and low SDECG scores correspond mainly to CI and CII of DEG clustering, respectively.

## 4 Discussion

### 4.1 TCM understanding of DN

DN belongs to the category of “Shuizhong” (edema), “Shenxiao” (renal diabetes), and “Guange” (obstruction and rejection) in TCM. According to TCM theory, this disease is characterized by weak viscera, moodiness, and excessive consumption of fatty and sweet foods. The root mechanism of DN is yin deficiency, and the tip mechanisms are dryness-heat and static blood. Therefore, TCM treatments that can tonify qi, nourish yin, and tonify kidney and spleen while promoting blood circulation and removing blood stasis are often used to treat DN. The medicinal materials used in TMJT are able to address these effects comprehensively.

### 4.2 The material basis of TMJT for treating DN

Through analysis of the “drug-component-target” network, we have identified the main material basis for the therapeutic effects of TMJT on DN. Denudatin B, a natural platelet antagonist, relaxes smooth muscle by inhibiting calcium ions (Ca^2+^) inward flow and increases the action of cyclic adenosine monophosphate (cAMP) to relax blood vessels. Platelets play a role in inflammation, coagulation, and fibrosis in the pathogenesis of DN, thus platelet antagonists have therapeutic effects on DN (40, 41). Hirudinoidine A, an ingredient extracted from leeches, has shown efficacy in the treatment of diabetic nephropathy by modulating inflammatory factors through lyophilized leech powder containing this ingredient (36). Gypenoside XXXV_qt and Gypenoside XXXVI_qt are the parent nucleus of Gypenoside-like ingredients, and their derivative Gypenoside XLIX has shown potential therapeutic effects on kidney injury through an insulin-like growth factor-binding protein 7 (IGFBP7)/insulin-like growth factor 1 receptor (IGF1R)-dependent mechanism (42). Isoflavanone, a type of isoflavonoid, has been shown to regulate blood glucose levels, reduce insulin resistance, and regulate inflammation and oxidative stress, playing an important role as a supplementary drug in treating DM and its complications (43). Quercetin, a flavonoid widely found in plants, has been shown to reverse the process of DN by reducing oxidative stress, fighting inflammation, and eliminating free radicals (44). Moupinamide has been reported to have anti-inflammatory effects, while inflammation plays a central role in the development and progression of DN (45, 46). Gypentonoside A_qt, the parent nucleus of Gypentonoside A, has shown significant activation of adenosine 5’-monophosphate-activated protein kinase (AMPK) phosphorylation, a key mechanism for regulating glucose, lipid, and energy metabolism in animal experiments (47). Myristic acid, as confirmed by Takato et al. (48), may reduce insulin-responsive glucose levels and body weight to improve hyperglycemia, making it a potential candidate for the prevention and treatment of type 2 DM and its related diseases. Bacunone has been found to ameliorate DN by inhibiting recombinant glycogen synthase kinase 3 beta (GSK-3β) activity to attenuate high glucose-induced oxidative damage in renal tubular duct epithelial cells of rat (NRK-52E) cells (49). Scropolioside A has been shown to modulate several inflammatory factors and exhibit anti-inflammatory activity in a study by Bas et al. (50), but further investigation is needed to determine its relationship with DN. Overall, most of the core components obtained from the network pharmacological analysis have direct or indirect therapeutic effects on DN.

### 4.3 Human sample validated TMJT targets for DN treatment

We first identified the core targets using the PPI network and then conducted a differential expression analysis of these targets in the GEO dataset to identify SDECGs. Our analysis identified several genes that have been previously implicated in the pathogenesis of DN. For instance, *SRC* encodes a non-receptor protein tyrosine kinase that has been shown to promote the development of DN by suppressing mitophagy (51). Similarly, *EGF*, a member of the epidermal growth factor superfamily, has been closely associated with DN and may serve as a biomarker of DN progression (52, 53). *GAPDH*, which encodes a phosphate dehydrogenase, has been linked to the pathogenesis of diabetic complications, including DN, by causing acute endothelial dysfunction (54). Cytokines such as *IL6* and *IL1B* have also been implicated in the pathogenesis of DN through increased vascular inflammation and fibrosis (55, 56). Simultaneously, we have identified a causal relationship between elevated IL1B levels and an increased risk of DN through MR analysis, providing a reference for future research into the mechanisms of DN onset.

Other SDECGs identified in our analysis include *VEGFA*, which is involved in abnormal angiogenesis in DN and has been shown to be a potential therapeutic target for DN (51), *CASP3*, which is involved in pyoptosis and has been suggested as a possible mechanism in the pathogenesis of DN (57, 58), *CTNNB1*, which regulates apoptosis in DN and is a potential therapeutic target (59), *MMP9*, which has been shown to play an important role in DN (60), *EP300*, which promotes the fibrotic process in renal tubular epithelial cells and contributes to the development and progression of DN (61), *PIK3R1*, which protects podocytes in DN by restoring autophagy and inhibiting apoptosis (62, 63), and *CD40LG*, which is involved in mediating the inflammatory response and remodeling associated with DN tissue damage and glomerulosclerosis (64).

Our differential expression analysis revealed that all the SDECGs except *CD40LG* were up-regulated in the DN group, which is consistent with previous studies. Additionally, we found that the SDECGs in DN mainly showed synergistic effects from the correlation analysis. It is noteworthy that a study (64) reports that the CD40LG/CD40 system is elevated in DM and its complications, including DN, because of its main manifestation as inflammation and remodeling. For this discrepancy, we think that first of all, the samples we used were peripheral blood samples from healthy and different stages of DN patients, which differed from the kidney tissue samples reported in the study; secondly, our sample size was relatively small, so the research results might be specific to certain sample size; moreover, although there is no report so far that *CD40LG* has bidirectional regulation or indirect effect in DN, our results suggested that *CD40LG* might have this effect, as shown by the correlation analysis of SDECGs that it was mainly positively correlated with other positively regulated SDECGs and negatively correlated with a few SDECGs. Therefore, the role of *CD40LG* in DN still needs further exploration and research.

### 4.4 Mechanism analysis of immune cells and pathways

The ssGSEA revealed statistically significant differences in the immune cell expression between the normal group and DN group, with comparable overall expression levels. Furthermore, our correlation analysis between the expression of SDECGs and immune cells revealed a statistically significant positive correlation between some of the SDECGs and immune cells. Among these immune cells that have statistical differences in expression: γδT cells have less research on their role in DN, and an experiment has shown that some T cell subsets are up-regulated in DN mice (65); NK cells are also up-regulated in the peripheral blood of DN patients in a retrospective clinical study, thus becoming a risk factor for DN (66); Dendritic cells also show up-regulation in both experimental and human studies of DN, and their mechanism may involve aspects such as high glucose, RAAS, cytokine secretion and proteinuria status (67). In addition, it should be pointed out that some studies have shown that M2 macrophage polarization has a positive correlation with renal fibrosis, which is a key pathological feature of DN progression (68). Our study also showed that M2 macrophages were positively correlated with two SDECGs with statistical significance, but there was no statistical difference in the differential analysis between DN and normal samples. The reason for this difference we think is firstly that the expression relationship between M2 macrophages and genes is different from the expression difference between M2 macrophages in normal and DN groups, which involves complex regulatory networks, thus resulting in inconsistency of results; and M2 macrophages only have regulatory relationship with 2/13 SDECGs, which may account for a small proportion in the level of SDECGs and DN relationship, thus being diluted in the dimension of disease gene expression and showing no statistical difference.

Additionally, we constructed a nomogram based on feature genes identified using SVM to quantitatively assess the onset of DN and the sensitivity and accuracy of TMJT in treating DN. We also validated the target screening and model construction by using molecular docking and the GEO dataset. By cluster analysis, our results were expanded, and the results indicated that the mechanisms of TMJT in treating DN were related to immune-related factors such as inflammation, oxidative stress, cytokines, and infection. These results are consistent with the current understanding that the pathogenesis of DN is mainly due to hyperglycemia leading to the expression of inflammatory mediators by damaged glomerular and tubular cells, which induce extracellular matrix deposition and myofibroblast differentiation/proliferation through different signaling pathways, thus leading to renal injury in different ways.

### 4.5 The strengths and limitations of this study

This study comprehensively explored the mechanisms of TMJT, a traditional Chinese medicine, in treating DN from multiple aspects, such as genes, proteins, immune cells, and pathways, based on various data of “drug-disease-human” and using methods such as network topology, machine learning, molecular docking, and MR. Previous studies (69, 70) on TMJT mainly focused on its safety and efficacy, while we conducted a mechanism analysis of TMJT based on this, filling the gap of the lack of mechanism exploration for this drug. Previous network pharmacology studies (71, 72) on traditional Chinese medicine or Chinese herbal medicine for treating DN did not involve in-depth analysis of human samples but used animal samples or simply collected disease targets from GEO datasets. Previous machine learning studies (73–75) on DN mainly analyzed it from the perspectives of immunity, oxidative stress, programmed cell death, etc., and were mainly used to screen and identify genetic biomarkers, while this study used the target as a link and started from the perspective of drug intervention in disease to explore the multi-faceted mechanisms of TMJT in treating DN, and verified the results by dataset analysis, molecular docking, MR analysis, and other methods. Therefore, compared with other studies, this study is more comprehensive and has more guiding significance for clinical medication.

However, further experiments and trials are needed to verify the accuracy of our results, as the information on drug components, targets, and patient samples in this study were obtained from public databases. Overall, our study provides valuable insights for clinical and future research on the therapeutic effects of TMJT on DN.

## 5 Conclusion

In conclusion, our study utilized a multi-faceted approach, combining network pharmacology, molecular docking, and bioinformatics, to analyze the components, targets, and mechanisms of TMJT in treating DN. Through this study, we identified the key components, targets, and feature genes of TMJT, including Denudatin B, hancinol, hirudinoidine A, *SRC*, *EGF*, *GAPDH*, *CD40LG*, *EP300*, *IL1B*, *and EGF*; We also confirmed that the elevation of *IL1B* levels has a causal relationship with the increased risk of DN. Our findings suggest that the therapeutic effects of TMJT on DN are primarily mediated through the regulation of immune and inflammation-related pathways. While these results are promising and useful as a reference for clinical application and further research, the accuracy of our findings should be verified through additional experiments and trials, particularly given that the data used in this study was obtained from public databases.

## Data availability statement

The datasets presented in this study can be found in online repositories. The names of the repository/repositories and accession number(s) can be found in the article/**Supplementary Material**.

## Author contributions

YL, XZ, ZX, and XC conceived the idea for the study. YL, XC and XZ retrieved the studies for inclusion and abstracted the data. YL, JX, WW, ZX, and YX performed the statistical analysis. JX, YL and YX interpreted the data. WW, YX critically revised the paper based on intellectual content. All authors contributed to the article and approved the submitted version.
