# Supplementary material for: Exploring the potential mechanisms of Tongmai Jiangtang capsules in treating diabetic nephropathy through multi-dimensional data
Source: Front Endocrinol (Lausanne). 2023 Nov 1;14:1172226. doi: 10.3389/fendo.2023.1172226 (PMC10654657; doi:10.3389/fendo.2023.1172226)
Supplement: Supplementary file 1 [file Table_1.pdf]

Table S1 Information of core components

MolID	MolName	OB(%)	DL	Herb
MOL000173	wogonin	30.68	0.23	Cangzhu
MOL000179	2-Hydroxyisoxypropyl-3-hydroxy-7-isopentene-2,3-dihydrobenzofuran-5-carboxylic	45.2	0.20	Cangzhu
MOL000184	NSC63551	39.25	0.76	Cangzhu
MOL000186	Stigmasterol 3-O-beta-D-glucopyranoside_qt	43.83	0.76	Cangzhu
MOL000188	3β-acetoxyatractylone	40.57	0.22	Cangzhu
MOL000085	beta-daucosterol_qt	36.91	0.75	Cangzhu
MOL000088	beta-sitosterol 3-O-glucoside_qt	36.91	0.75	Cangzhu
MOL000092	daucosterin_qt	36.91	0.76	Cangzhu
MOL000094	daucosterol_qt	36.91	0.76	Cangzhu
MOL001601	1,2,5,6-tetrahydrotanshinone	38.75	0.36	Danshen
MOL001659	Poriferasterol	43.83	0.76	Danshen
MOL001771	poriferast-5-en-3beta-ol	36.91	0.75	Danshen, Dongkuiguo
MOL001942	isoimperatorin	45.46	0.23	Danshen
MOL002222	sugiol	36.11	0.28	Danshen, Xuanshen
MOL002651	Dehydrotanshinone II A	43.76	0.40	Danshen
MOL002776	Baicalin	40.12	0.75	Danshen
MOL000569	digallate	61.85	0.26	Danshen
MOL000006	luteolin	36.16	0.25	Danshen, Dongkuiguo, Taizishen
MOL007036	5,6-dihydroxy-7-isopropyl-1,1-dimethyl-2,3-dihydrophenanthren-4-one	33.77	0.29	Danshen
MOL007041	2-isopropyl-8-methylphenanthrene-3,4-dione	40.86	0.23	Danshen
MOL007045	3α-hydroxytanshinoneIIa	44.93	0.44	Danshen
MOL007048	(E)-3-[2-(3,4-dihydroxyphenyl)-7-hydroxy-benzofuran-4-yl]acrylic acid	48.24	0.31	Danshen
MOL007049	4-methylenemiltirone	34.35	0.23	Danshen
MOL007050	2-(4-hydroxy-3-methoxyphenyl)-5-(3-hydroxypropyl)-7-methoxy-3-benzofurancarboxaldehyde	62.78	0.40	Danshen
MOL007058	formyltanshinone	73.44	0.42	Danshen
MOL007059	3-beta-Hydroxymethyllenetanshiquinone	32.16	0.41	Danshen
MOL007061	Methylenetanshinquinone	37.07	0.36	Danshen
MOL007063	przewalskin a	37.11	0.65	Danshen
MOL007064	przewalskin b	110.32	0.44	Danshen
MOL007068	Przewaquinone B	62.24	0.41	Danshen
MOL007069	przewaquinone c	55.74	0.40	Danshen
MOL007070	(6S,7R)-6,7-dihydroxy-1,6-dimethyl-8,9-dihydro-7H-naphtho[8,7-g]benzofuran-10,11-dione	41.31	0.45	Danshen
MOL007071	przewaquinone f	40.31	0.46	Danshen
MOL007077	sclareol	43.67	0.21	Danshen
MOL007079	tanshinaldehyde	52.47	0.45	Danshen

MOL007081	Danshenol B	57.95	0.56	Danshen
MOL007082	Danshenol A	56.97	0.52	Danshen
MOL007085	Salvilenone	30.38	0.38	Danshen
MOL007088	cryptotanshinone	52.34	0.40	Danshen
MOL007093	dan-shexinkum d	38.88	0.55	Danshen
MOL007094	danshenspiroketallactone	50.43	0.31	Danshen
MOL007098	deoxyneocryptotanshinone	49.40	0.29	Danshen
MOL007100	dihydrotanshinlactone	38.68	0.32	Danshen
MOL007101	dihydrotanshinoneI	45.04	0.36	Danshen
MOL007105	epidanshenspiroketallactone	68.27	0.31	Danshen
MOL007107	C09092	36.07	0.25	Danshen
MOL007108	isocryptotanshi-none	54.98	0.39	Danshen
MOL007111	Isotanshinone II	49.92	0.40	Danshen
MOL007115	manool	45.04	0.20	Danshen
MOL007119	miltionone I	49.68	0.32	Danshen
MOL007120	miltionone II	71.03	0.44	Danshen
MOL007121	miltipolone	36.56	0.37	Danshen
MOL007122	Miltirone	38.76	0.25	Danshen
MOL007124	neocryptotanshinone ii	39.46	0.23	Danshen
MOL007125	neocryptotanshinone	52.49	0.32	Danshen
MOL007127	1-methyl-8,9-dihydro-7H-naphtho[5,6-g]benzofuran-6,10,11-trione	34.72	0.37	Danshen
MOL007130	prolithospermic acid	64.37	0.31	Danshen
MOL007132	(2R)-3-(3,4-dihydroxyphenyl)-2-[(Z)-3-(3,4-dihydroxyphenyl)acryloyl]oxy-propionic acid	109.38	0.35	Danshen
MOL007141	salvianolic acid g	45.56	0.61	Danshen
MOL007142	salvianolic acid j	43.38	0.72	Danshen
MOL007143	salvilenone I	32.43	0.23	Danshen
MOL007145	salviolone	31.72	0.24	Danshen
MOL007150	(6S)-6-hydroxy-1-methyl-6-methylol-8,9-dihydro-7H-naphtho[8,7-g]benzofuran-10,11-quinone	75.39	0.46	Danshen
MOL007151	Tanshindiol B	42.67	0.45	Danshen
MOL007152	Przewaquinone E	42.85	0.45	Danshen
MOL007154	tanshinone iia	49.89	0.40	Danshen
MOL007155	(6S)-6-(hydroxymethyl)-1,6-dimethyl-8,9-dihydro-7H-naphtho[8,7-g]benzofuran-10,11-dione	65.26	0.45	Danshen
MOL007156	tanshinone VI	45.64	0.30	Danshen
MOL006824	α-amyrin	39.51	0.76	Danshen
MOL007051	6-o-syringyl-8-o-acetyl shanzhiside methyl ester	46.69	0.71	Danshen
MOL007118	microstegiol	39.61	0.28	Danshen
MOL007123	miltirone II	44.95	0.24	Danshen
MOL007140	(Z)-3-[2-[(E)-2-(3,4-dihydroxyphenyl)vinyl]-3,4-dihydroxy-phenyl]acrylic acid	88.54	0.26	Danshen
MOL007149	NSC 122421	34.49	0.28	Danshen
MOL000358	beta-sitosterol	36.91	0.75	Dongkuiguo, Gegen, Taizishen,

				Xuanshen
MOL008334	8-quinolyl octadecanoate	44.79	0.59	Dongkuiguo
MOL000098	quercetin	46.43	0.28	Dongkuiguo, Huanglian, Huangqi, Jiaogulan
MOL000392	formononetin	69.67	0.21	Gegen, Huangqi
MOL002959	3'-Methoxydaidzein	48.57	0.24	Gegen
MOL003629	Daidzein-4,7-diglucoside	47.27	0.67	Gegen
MOL001454	berberine	36.86	0.78	Huanglian
MOL002894	berberrubine	35.74	0.73	Huanglian
MOL002897	epiberberine	43.09	0.78	Huanglian
MOL002903	(R)-Canadine	55.37	0.77	Huanglian
MOL002904	Berlambine	36.68	0.82	Huanglian
MOL002907	Corchoroside A_qt	104.95	0.78	Huanglian
MOL000622	Magnograndiolide	63.71	0.19	Huanglian
MOL000098	quercetin	46.43	0.28	Huanglian
MOL001458	coptisine	30.67	0.86	Huanglian
MOL002668	Worenine	45.83	0.87	Huanglian
MOL013352	Obacunone	43.29	0.77	Huanglian
MOL000762	Palmidin A	35.36	0.65	Huanglian
MOL008647	Moupinamide	86.71	0.26	Huanglian
MOL000211	Mairin	55.38	0.78	Huangqi
MOL000239	Jaranol	50.83	0.29	Huangqi
MOL000296	hederagenin	36.91	0.75	Huangqi
MOL000033	(3S,8S,9S,10R,13R,14S,17R)-10,13-dimethyl-17-[(2R,5S)-5-propan-2-yloctan-2-yl]-2,3,4,7,8,9,11,12,14,15,16,17-dodecahydro-1H-cyclopenta[a]phenanthren-3-ol	36.23	0.78	Huangqi
MOL000354	isorhamnetin	49.6	0.31	Huangqi
MOL000371	3,9-di-O-methylnissolin	53.74	0.48	Huangqi
MOL000378	7-O-methylisomucronulatol	74.69	0.30	Huangqi
MOL000379	9,10-dimethoxypterocarpan-3-O-β-D-glucoside	36.74	0.92	Huangqi
MOL000380	(6aR,11aR)-9,10-dimethoxy-6a,11a-dihydro-6H-benzofurano[3,2-c]chromen-3-ol	64.26	0.42	Huangqi
MOL000387	Bifendate	31.1	0.67	Huangqi
MOL000417	Calycosin	47.75	0.24	Huangqi
MOL000422	kaempferol	41.88	0.24	Huangqi
MOL000433	FA	68.96	0.71	Huangqi
MOL000439	isomucronulatol-7,2'-di-O-glucosiole	49.28	0.62	Huangqi
MOL000442	1,7-Dihydroxy-3,9-dimethoxy pterocarpene	39.05	0.48	Huangqi
MOL000374	5'-hydroxyiso-muronulatol-2',5'-di-O-glucoside	41.72	0.69	Huangqi
MOL000398	isoflavanone	109.99	0.30	Huangqi
MOL000438	(3R)-3-(2-hydroxy-3,4-dimethoxyphenyl)chroman-7-ol	67.67	0.26	Huangqi
MOL000338	3'-methyleriodictyol	51.61	0.27	Jiaogulan
MOL000351	Rhamnazin	47.14	0.34	Jiaogulan

MOL000359	sitosterol	36.91	0.75	Jiaogulan, Xuanshen
MOL004350	Ruvoside_qt	36.12	0.76	Jiaogulan
MOL004355	Spinasterol	42.98	0.76	Jiaogulan
MOL005438	campesterol	37.58	0.71	Jiaogulan
MOL005440	Isofucosterol	43.78	0.76	Jiaogulan
MOL000953	CLR	37.87	0.68	Jiaogulan, Shanyao
MOL009855	(24S)-Ethylcholesta-5,22,25-trans-3beta-ol	46.91	0.76	Jiaogulan
MOL009867	4α,14α-dimethyl-5α-ergosta-7,9(11),24(28)-trien-3β-ol	46.29	0.76	Jiaogulan
MOL009877	cucurbita-5,24-dienol	44.02	0.74	Jiaogulan
MOL009878	Cyclobuxine	84.48	0.70	Jiaogulan
MOL009971	Gypenoside XXVII_qt	30.21	0.74	Jiaogulan
MOL009973	Gypenoside XXVIII_qt	32.08	0.74	Jiaogulan
MOL007475	ginsenoside f2	36.43	0.25	Jiaogulan
MOL009888	Gypenoside XXXVI_qt	37.85	0.78	Jiaogulan
MOL009928	Gypenoside LXXIV	34.21	0.24	Jiaogulan
MOL009929	Gypenoside LXXIX	37.75	0.25	Jiaogulan
MOL009938	Gypenoside XII	36.43	0.25	Jiaogulan
MOL009943	Gypenoside XL	30.89	0.21	Jiaogulan
MOL009969	Gypenoside XXXV_qt	37.73	0.78	Jiaogulan
MOL009976	Gypenoside XXXII	34.24	0.25	Jiaogulan
MOL009986	Gypentonoside A_qt	36.13	0.80	Jiaogulan
MOL001559	piperlonguminine	30.71	0.18	Shanyao
MOL001736	(-)-taxifolin	60.51	0.27	Shanyao
MOL000322	Kadsurenone	54.72	0.38	Shanyao
MOL005430	hancinone C	59.05	0.39	Shanyao
MOL005435	24-Methylcholest-5-enyl-3belta-O-glucopyranoside_qt	37.58	0.72	Shanyao
MOL000449	Stigmasterol	43.83	0.76	Shanyao
MOL005458	Dioscoreside C_qt	36.38	0.87	Shanyao
MOL000546	diosgenin	80.88	0.81	Shanyao
MOL005465	AIDS180907	45.33	0.77	Shanyao
MOL000310	Denudatin B	61.47	0.38	Shanyao
MOL005429	hancinol	64.01	0.37	Shanyao
MOL005461	Doradexanthin	38.16	0.54	Shanyao
MOL005463	Methylcimicifugoside_qt	31.69	0.24	Shanyao
MOL004469	2-Piperidone	82.45	0.02	Shuizhi
MOL001788	adenine	62.81	0.03	Shuizhi
MOL001732	glycerine	72.87	0.01	Shuizhi
N1	hirudinoidine A	High	4*yes	Shuizhi
MOL001831	hypoxanthine	52.29	0.04	Shuizhi
MOL000421	Nicotinic acid	47.65	0.02	Shuizhi
MOL000069	palmitic acid	19.30	0.10	Shuizhi
MOL000346	Succinic acid	29.62	0.07	Shuizhi
MOL001744	uracil	42.53	0.02	Shuizhi
MOL010716	xanthine	46.17	0.02	Shuizhi
N2	1-O-Palmityl-rac-glycerol	High	3*yes	Shuizhi

MOL000041	phenylalanine	41.62	0.04	Shuizhi
N3	propylamine	High	3*yes	Shuizhi
MOL000061	proline	77.57	0.01	Shuizhi
MOL000067	valine	53.33	0.01	Shuizhi
MOL000068	L-isoleucine	59.05	0.02	Shuizhi
MOL000666	Hexanal	55.71	0.01	Shuizhi
N4	Methyl 4-methyltetradecanoate	High	3*yes	Shuizhi
MOL001393	Myristic acid	21.18	0.07	Shuizhi
N5	Methyl 14-methylpentadecanoate	High	3*yes	Shuizhi
N6	zinc	N/A	N/A	Shuizhi
N7	Molybdenum	N/A	N/A	Shuizhi
N8	magnesium	N/A	N/A	Shuizhi
N9	copper	N/A	N/A	Shuizhi
N10	calcium	N/A	N/A	Shuizhi
MOL001506	Supraene	33.55	0.42	Taizishen
MOL001689	acacetin	34.97	0.24	Taizishen
MOL001790	Linarin	39.84	0.71	Taizishen
MOL006554	Taraxerol	38.40	0.77	Taizishen
MOL006756	Schottenol	37.42	0.75	Taizishen
MOL002464	1-Monolinolein	37.18	0.30	Taizishen
MOL007658	14-deoxy-12(R)-sulfoandrographolide	62.57	0.42	Xuanshen
MOL007662	harpagoside_qt	122.87	0.32	Xuanshen
MOL001925	paeoniflorin_qt	68.18	0.40	Xuanshen
MOL007657	scropolioside A_qt	38.63	0.77	Xuanshen
MOL007659	scropolioside D	36.62	0.40	Xuanshen
MOL007660	scropolioside D_qt	33.17	0.82	Xuanshen

Table S2 Binding energy of molecular docking

Unit:kcal/mol

MOLID	MOLNAME	Target PDBID	EGF 1IVO	GAPDH 4WNC	EP300 4PZR	IL1B 1HIb	CD40LG 7sgm
MOL000310	Denudatin B		-5.3	-8.3	-8.3	-6.2	-7.5
MOL005429	hancinol		-5.5	-8.2	-7.8	-6.0	-7.1
NA1	hirudinoidine A		-6.1	-9.1	-8.8	-7.3	-7.5
MOL009969	Gypenoside XXXV_qt		-6.9	-10.5	-9.1	-7.0	-8.1
MOL000398	isoflavanone		-6.6	-10.1	-9.3	-7.9	-8.2
MOL000098	quercetin		-6.3	-9.1	-8.9	-7.1	-7.4
MOL008647	Moupinamide		-6.0	-8.7	-9.2	-6.8	-7.9
MOL009986	Gypentonoside A_qt		-6.2	-9.0	-9.9	-7.2	-7.9
MOL000438	(3R)-3-(2-hydroxy-3,4-dimethoxyphenyl)chroman-7-ol		-6.6	-9.8	-9.1	-6.9	-8.0
MOL001393	Myristic acid		-6.0	-8.4	-8.2	-6.3	-6.9
MOL007123	miltirone II		-6.3	-9.4	-9.6	-7.0	-7.4
MOL013352	Obacunone		-7.3	-9.9	-11.5	-8.7	-9.2
MOL000179	2-Hydroxyisoxypropyl-3-hydroxy-7-isopentene-2,3-dihydrobenzofuran-5-carboxylic		-6.6	-9.9	-8.5	-7.1	-8.5
MOL000069	palmitic acid		-3.9	-5.5	-6.2	-4.2	-4.7
NA4	Methyl 4-methyltetradecanoate		-5.0	-5.9	-6.4	-5.2	-5.6
MOL009888	Gypenoside XXXVI_qt		-6.8	-10.3	-10.9	-7.9	-9.2
MOL007657	scropolioside A_qt		-7.9	-12.3	-10.9	-9.5	-9.8

Table S3 SNPs information for the 5 feature genes

Gene	SNP	A1	A2	Chr	n	beta	se	p	eaf	pos.exposure	R2	F value
	rs3129934	C	T	6	7715	-0.08279	0.016478	5.06E-07	0.846073	32336187	0.003261	25.23588
CD40LG	rs2894207	C	T	6	8491	-0.06844	0.014926	4.53E-06	0.197958	31263751	0.00247	21.01992
	rs2247056	C	T	6	8491	-0.0826	0.013792	2.11E-09	0.753447	31265490	0.004207	35.86333
	rs10408945	T	G	19	9188	0.078616	0.017021	3.86E-06	0.142342	16576991	0.002316	21.32776
	rs12166430	G	A	22	31233	-0.10051	0.020589	1.05E-06	0.091865	42294176	0.000762	23.82954
	rs76120255	A	C	22	14338	-0.22293	0.048603	4.50E-06	0.015204	41906827	0.001465	21.03491
	rs13058113	T	G	22	31567	0.361734	0.013064	9.68E-169	0.269459	41508414	0.023711	766.606
	rs117597170	A	G	22	26101	0.357775	0.03493	1.28E-24	0.029701	41545623	0.004003	104.9046
	rs142143747	A	G	22	27004	-0.26221	0.031395	6.71E-17	0.037143	41602148	0.002576	69.74939
	rs116962367	G	A	22	26897	0.321415	0.041103	5.30E-15	0.021332	42034891	0.002268	61.14328
	rs184725464	A	G	22	25655	0.334203	0.037078	2.00E-19	0.026312	41411567	0.003157	81.23882
	rs71327105	G	T	22	27060	-0.17528	0.027384	1.55E-10	0.049557	41410124	0.001512	40.96608
	rs2235851	A	G	22	31484	-0.14296	0.015423	1.87E-20	0.180658	41660645	0.002722	85.91749
EP300	rs133077	T	C	22	31567	-0.16219	0.012315	1.30E-39	0.36126	41084635	0.005465	173.4459
	rs116856246	A	G	22	22309	-0.28279	0.044437	1.97E-10	0.01822	41500750	0.001812	40.4956
	rs117072685	A	G	22	26894	0.300398	0.03033	3.99E-23	0.039828	41518460	0.003634	98.0876
	rs117705111	A	G	22	27068	0.348249	0.035087	3.23E-23	0.029441	41957510	0.003626	98.5043
	rs112045239	A	G	22	31597	0.117583	0.019608	2.01E-09	0.102387	42329284	0.001137	35.95906
	rs138330	G	C	22	22702	0.218309	0.039586	3.49E-08	0.97691	41215737	0.001338	30.41038
	rs2267442	A	G	22	31560	0.063764	0.012274	2.05E-07	0.623787	42280361	0.000854	26.98549
	rs148621721	T	C	22	24532	-0.216	0.042321	3.33E-07	0.020147	41467696	0.001061	26.04751
	rs9611560	C	T	22	31346	0.188491	0.014004	2.70E-41	0.76771	41750622	0.005746	181.1468
	rs4820415	A	G	22	26419	0.152993	0.031752	1.45E-06	0.036404	41025759	0.000878	23.21541

	rs76653253	T	C	22	28786	0.253082	0.033723	6.16E-14	0.032053	41038054	0.001953	56.31806
	rs117389145	A	G	22	24812	-0.23716	0.028104	3.21E-17	0.046816	41350824	0.002862	71.20278
	rs75066103	G	T	22	25455	0.316297	0.034758	9.03E-20	0.030054	41512154	0.003243	82.80498
	rs111355898	A	G	22	26659	-0.30261	0.019046	7.65E-57	0.107487	41530438	0.00938	252.4119
	rs118070243	C	A	22	24485	-0.26164	0.034641	4.25E-14	0.030319	41816110	0.002324	57.04021
	rs6542095	T	C	2	31183	-0.06013	0.012983	3.63E-06	0.700379	113529183	0.000687	21.45133
	rs115171104	T	C	2	14479	0.228108	0.04284	1.01E-07	0.019649	114037780	0.001954	28.34838
	rs6542082	G	A	2	30902	-0.09655	0.011884	4.51E-16	0.481182	113464709	0.002131	65.99327
	rs3917386	C	T	2	25820	0.14867	0.032055	3.52E-06	0.035695	113579866	0.000832	21.50853
	rs28498283	T	A	2	26953	-0.06777	0.013655	6.93E-07	0.254318	43360065	0.000913	24.6295
	rs1531070	A	G	4	31684	-0.07398	0.012069	8.81E-10	0.413713	140795327	0.001184	37.56819
	rs6893300	A	C	5	31684	-0.08158	0.013003	3.51E-10	0.297362	179135815	0.001241	39.36328
	rs149110519	T	C	6	25735	-0.19499	0.026607	2.33E-13	0.052616	144385777	0.002082	53.69951
IL1B	rs56388170	T	G	7	31355	0.087407	0.013107	2.58E-11	0.289189	28724374	0.001416	44.47142
	rs149007767	T	C	7	30077	0.165055	0.016624	3.12E-23	0.149708	50370254	0.003267	98.57643
	rs7846314	T	A	8	31569	-0.08826	0.01553	1.32E-08	0.178349	61650831	0.001022	32.29567
	rs4149577	A	G	12	31684	-0.05938	0.011926	6.40E-07	0.537074	6447522	0.000782	24.78582
	rs2038255	T	C	14	30165	0.096665	0.015579	5.48E-10	0.176825	35559126	0.001275	38.49847
	rs111778408	A	G	14	30165	-0.16672	0.019721	2.81E-17	0.100785	35572163	0.002364	71.467
	rs2290400	C	T	17	31300	-0.08048	0.011885	1.27E-11	0.493155	38066240	0.001463	45.84913
	rs8078723	C	T	17	31684	0.130981	0.012213	7.77E-27	0.379928	38166879	0.003617	115.018
	rs12478601	T	C	2	21602	-0.05949	0.012028	7.59E-07	0.574604	43721508	0.001131	24.45786
	rs10098310	A	G	8	21986	-0.05823	0.01203	1.30E-06	0.575007	130613614	0.001064	23.42425
GAPDH	rs12427370	A	G	12	15546	0.058875	0.012147	1.25E-06	0.398508	6538309	0.001509	23.4912
	rs2534725	C	T	12	15754	0.113058	0.018391	7.88E-10	0.118491	6557742	0.002393	37.7861
	rs73044244	T	C	12	15147	-0.42346	0.049544	1.26E-17	0.014569	6620226	0.0048	73.04516

	rs146094919	A	G	12	18122	0.186009	0.035905	2.21E-07	0.028222	6662485	0.001479	26.83568
	rs146648385	G	A	12	17604	-0.24658	0.045447	5.77E-08	0.017418	6873093	0.001669	29.43478
	rs2286725	T	C	12	20778	-0.34051	0.037968	3.01E-19	0.025062	6658860	0.003856	80.42247
	rs2267965	C	T	12	17065	0.103881	0.012467	7.94E-17	0.347657	6556946	0.004052	69.42093
	rs78187632	C	T	12	21348	0.345157	0.034941	5.17E-23	0.029698	6675870	0.00455	97.57184
	rs55677006	G	C	12	18915	-0.08818	0.017984	9.43E-07	0.124952	6826159	0.001269	24.03777
	rs146805151	C	T	12	16155	0.158382	0.031003	3.25E-07	0.038249	6538217	0.001613	26.09459
	rs73047887	C	T	12	21572	0.114435	0.013879	1.65E-16	0.2412	6736843	0.003142	67.9798
	rs10774442	T	C	12	21657	-0.08209	0.013235	5.56E-10	0.719825	6829472	0.001773	38.46896
	rs150807305	A	G	12	17613	-0.39977	0.043089	1.73E-20	0.019337	6543669	0.004863	86.06565
	rs11569357	C	T	12	20580	-0.25878	0.024196	1.07E-26	0.064134	6553336	0.005528	114.3807
	rs78312001	G	C	12	21400	0.246551	0.035608	4.39E-12	0.028665	6744996	0.002235	47.93723
	rs117471807	C	T	12	20355	0.218366	0.036186	1.60E-09	0.027754	6785945	0.001786	36.41295
	rs80106965	G	T	12	19560	-0.37694	0.028149	6.79E-41	0.046289	6807536	0.009085	179.3054
	rs9634157	G	A	12	21648	-0.48257	0.013789	1.00E-200	0.219682	6648198	0.053545	1224.605
	rs11064382	A	G	12	20494	-0.08413	0.0179	2.60E-06	0.12635	6891195	0.001077	22.08603
	rs11064245	A	G	12	21986	0.231666	0.013197	5.49E-69	0.274289	6631725	0.013823	308.1398
	rs73044250	T	C	12	17081	-0.24077	0.045836	1.50E-07	0.017121	6624733	0.001613	27.58871
	rs13098914	T	C	3	26229	0.056461	0.01205	2.79E-06	0.580178	56834799	0.000836	21.95223
	rs1354034	C	T	3	31684	0.288771	0.012007	8.18E-128	0.618137	56849749	0.017929	578.3959
	rs17825630	A	G	3	31684	0.12985	0.019135	1.15E-11	0.108121	56950055	0.001451	46.04681
EGF	rs9714929	C	T	4	31559	-0.06712	0.01405	1.78E-06	0.766161	110675356	0.000723	22.81818
	rs112835441	C	T	4	25213	0.144009	0.031308	4.23E-06	0.037491	110686307	0.000838	21.15644
	rs80008037	A	G	4	25906	-0.11013	0.019912	3.19E-08	0.098939	110529953	0.001179	30.58531
	rs2298979	A	G	4	31684	0.242905	0.011953	8.12E-92	0.404638	110838126	0.012867	412.979
	rs3756261	C	T	4	31684	0.303242	0.024634	8.04E-35	0.061539	110832306	0.00476	151.5191

rs75433216	C	A	4	31240	0.131637	0.025096	1.56E-07	0.059698	111037249	0.00088	27.51137
rs74989581	T	C	4	30873	0.142052	0.023395	1.26E-09	0.069366	110980201	0.001193	36.86679
rs75946500	C	A	4	26892	-0.09208	0.012583	2.52E-13	0.664665	110797089	0.001988	53.55081
rs7683007	G	C	4	31562	0.095329	0.012819	1.03E-13	0.312229	110982130	0.001749	55.30235
rs4141077	G	T	4	29164	-0.18166	0.038683	2.65E-06	0.024223	110883341	0.000756	22.05287
rs113097224	C	T	4	28473	-0.14913	0.023365	1.74E-10	0.069536	110837520	0.001429	40.73592
rs9399137	C	T	6	31569	0.071272	0.013482	1.25E-07	0.264393	135419018	0.000884	27.94319
rs113422568	A	G	10	27738	-0.076	0.013212	8.80E-09	0.28197	104337938	0.001191	33.08567
rs7075195	G	A	10	31569	0.062396	0.012043	2.20E-07	0.421253	65050659	0.00085	26.84408
rs505404	G	T	11	31569	0.104314	0.014294	2.92E-13	0.222031	243268	0.001684	53.25747
rs79755767	A	G	12	30744	0.117184	0.020018	4.80E-09	0.097736	54698408	0.001113	34.26594
rs11553699	G	A	12	30031	0.130589	0.019018	6.58E-12	0.10963	122216910	0.001568	47.14606
rs10512472	C	T	17	31684	0.079561	0.01551	2.90E-07	0.179037	33884804	0.00083	26.31221
rs1615504	C	T	18	31569	0.056694	0.011904	1.91E-06	0.520457	67526644	0.000718	22.68099

Table S4 Results of MR Analysis of feature genes and DN

Gene	ID number	MR Methods	N SNPs	OR (95%CI)	*P* value
		MR Egger	4	6.69E-10(2.11E-22 ,2115.09)	2.87E-01
		Weighted median	4	1.34(0.71 ,2.51)	3.66E-01
CD40LG	eqtl-a-ENSG00000102245	IVW	4	0.78(0.07, 9.26)	8.47E-01
		Simple mode	4	1.69(0.83 ,3.44)	2.45E-01
		Weighted mode	4	1.40(0.76 ,2.56)	3.56E-01
		MR Egger	23	1.10(0.88 ,1.38)	4.05E-01
		Weighted median	23	0.97(0.86 ,1.10)	6.36E-01
EP300	eqtl-a-ENSG00000100393	IVW	23	0.95(0.86 ,1.05)	2.93E-01
		Simple mode	23	1.02(0.84 ,1.25)	8.13E-01
		Weighted mode	23	0.99(0.87 ,1.13)	8.59E-01
		MR Egger	16	1.54(0.99 ,2.40)	7.62E-02
		Weighted median	16	1.29(1.05 ,1.59)	1.57E-02
IL1B	eqtl-a-ENSG00000125538	IVW	16	1.23(1.06 ,1.43)	6.88E-03
		Simple mode	16	1.38(0.97 ,1.96)	9.72E-02
		Weighted mode	16	1.32(1.01 ,1.71)	5.70E-02
		MR Egger	20	0.90(0.77 ,1.05)	1.87E-01
		Weighted median	20	0.96(0.86 ,1.07)	4.21E-01
GAPDH	eqtl-a-ENSG00000111640	IVW	20	0.95(0.87 ,1.05)	3.04E-01
		Simple mode	20	0.96(0.77 ,1.18)	6.92E-01
		Weighted mode	20	0.95(0.85 ,1.06)	3.96E-01
		MR Egger	21	1.13(0.93 ,1.38)	2.45E-01
EGF	eqtl-a-ENSG00000138798	Weighted median	21	1.04(0.91 ,1.18)	5.94E-01
		IVW	21	1.00(0.90 ,1.11)	9.81E-01

	Simple mode	21	0.94(0.74 ,1.18)	5.87E-01
	Weighted mode	21	1.04(0.91 ,1.18)	5.82E-01

Table S5 Heterogeneity and Pleiotropy of MR Analysis results

Gene	Heterogeneity				Pleiotropy		MR-PRESSO	
	MR Egger		IVW		MR Egger		Global Test	
	Cochran's Q	P-value	Cochran's Q	P-value	Egger intercept	P-value	RSSobs	P-value
CD40LG	46.94	6.41E-11	94.67	2.17E-20	1.64	0.29	156.90	＜0.001
EP300	30.13	0.09	33.21	0.06	-0.04	0.16	35.95	0.075
IL1B	9.18	0.82	10.30	0.80	-0.02	0.31	11.44	0.833
GAPDH	25.36	0.12	26.60	0.11	0.016	0.36	31.68	0.116
EGF	14.96	0.72	17.00	0.65	-0.020	0.17	18.26	0.694
